# Comprehensive Characterization of Aroma Profile of “Glutinous Rice” Flavor in *Pandanus amaryllifolius Roxb.* Using HS–SPME–GC–O–MS and HS-GC-IMS Technology Coupled with OAV

**DOI:** 10.3390/foods14060935

**Published:** 2025-03-10

**Authors:** Kun Tang, Cong Chen, Yutong Liu, Suxuan Li, Yiye Luo, Xiaoyu Chen, Zhiyong Wu

**Affiliations:** Flavors and Fragrance Engineering & Technology Research Center of Henan Province, College of Tobacco Science, Henan Agricultural University, Zhengzhou 450046, China; tangk135@163.com (K.T.); cc1822043@163.com (C.C.); l728965256@163.com (Y.L.); lsx991126@163.com (S.L.); 19838107782@163.com (Y.L.); xiaoyuchen@henau.edu.cn (X.C.)

**Keywords:** HS-SPME-GC-O-MS, HS-GC-IMS, *Pandanus amaryllifolius Roxb*., drying method, aroma volatiles, odor activity value

## Abstract

Pandan leaves have a prominent glutinous-rice aroma; however, few studies have explored their volatile aroma compound composition. Herein, the differences in the volatile aroma components of fresh and dried pandan leaves were investigated for the first time using HS–SPME–GC–O–MS combined with principal component analysis, orthogonal partial least squares discriminant analysis, and HS-GC-IMS with aroma fingerprinting. A total of 93 volatile compounds were identified, exceeding previous reports, including 43 main flavor components with odor activity values (OAV) > 1. OAV and aroma extract dilution analysis tests reveal 13 main aroma volatiles including 2-acetyl-1-pyrroline, hexanal, nonanal, phenylacetaldehyde, β-cyclocitral, butanal, ethyl caprylate, ethyl nonanoate, ethyl caprate, ethyl laurate, 3-hydroxy-2-butanone, acetophenone, and α-ionone. Sixteen types of aromas were classified, and the results are presented as flavor wheels. The findings of this study elucidate the changes and retention of aroma volatiles in differently processed leaves, which could benefit food industry applications.

## 1. Introduction

*Pandanus amaryllifolius Roxb.* or “pandan”, which belongs to the *Pandanaceae* family, is a tropical plant of the Pandanus (screw pine) genus. Pandan is the only Pandanus species with fragrant leaves and is primarily distributed throughout Southeast Asia, including Malaysia, Thailand, Indonesia, and China. Pandan leaves have a “sticky rice” fragrance and are known as the “vanilla of the Orient” [1]. Thus, pandan leaves are primarily used in the folk and spice industries as a spice additive or flavor adjunct.

Pandan-leaf processing is an important aspect of industrial production. Owing to the considerable loss of aroma and increased perishability caused by moisture in pandan leaves, common pre-treatments such as dehydration, thermal processing, and freeze-drying are employed. However, the flavor, preservation, and transport quality of food products are significantly affected by such processing treatments. For example, dehydration extends food-product shelf life while also inducing new attractive organoleptic properties; thermal processing applies high temperatures that induce the release of new aroma substances, thereby rendering the food more flavorful; and freeze-drying preserves food by reducing water activity and chemical reaction rates, while also having the potential to yield a higher recovery of key aroma components, which can provide a more pronounced characteristic flavor. However, freeze-drying slightly reduces the proportion of volatile or aroma-active compounds. To maximize pandan-leaf aroma, spray drying [2], solvothermal extraction [3], and freeze-drying [4] have been used for pandan-leaf processing; however, different processing techniques yield significant differences in the green note and mild pandan aroma of the resultant pandan leaves [5,6].

Pandan-leaf oil comprises various volatile compounds encompassing groups of alcohols, aliphatic compounds, carboxylic acids, ketones, aldehydes, esters, hydrocarbons, furans, furanones, and terpenoids [4]. Several studies have analyzed the organoleptic origins of the “glutinous rice” flavor of pandan leaves, focusing on the volatile compounds responsible for this characteristic flavor. Many studies have focused on 2-acetyl-1-pyrroline (2-AP) [1,3,7], which is one of the main sources of the characteristic pandan-leaf aroma. In the 1970s, researchers conducted an extraction of pandan leaves with a blend of chloroform and methanol as the solvent mixture, followed by the use of thin-layer chromatography to identify an oxidized product that included a yellow carotenoid pigment [8], and this polymer was described as peculiarly popcorn-like, with a typical pandan-leaf aroma. Buttery reported using vacuum water-vapor distillation in sequential extraction combined with gas chromatography–mass spectrometry (GC-MS) to identify a major eluting component in 12 volatile oils obtained from lyophilized samples. The Kovats retention index (KI) of this component matched that of synthesized 2-AP samples [9]. He also found that 2-AP was ten times more abundant in pandan leaves than in aromatic rice, indicating that 2-AP is not only the main differential flavoring substance among different aromatic rice varieties but also a compound that contributes significantly to the flavor of pandan leaves.

In later years, pre-treatment measures were primarily used for in-depth investigation of other volatile aroma components of pandan leaves, including supercritical CO_2_ extraction [10,11], solvent extraction [12,13], and simultaneous distillation-extraction combined with GC-MS. For example, Jiang has performed the centrifugal extraction of fresh pandan-leaf samples using dichloromethane as a solvent and analyzed the supernatant in combination with GC-MS and obtained 22 compounds [14]. The results indicated that 3-methyl-2(5H)-furanone was the major extract component, constituting 73% of the total volatile components, which were mainly characterized as having a caramelized, herbaceous aroma. Other major volatile components included 3-hexanol, 4-methyl-2-pentanol, 3-hexanone, and 2-hexanone, collectively accounting for 2.65–7.09% of the total volatile profile. These researchers concluded that C6 compounds contribute significantly to the fresh, green aroma profile of pandan leaves. Wakte identified the volatile constituents of 37 different varieties of pandan leaves distributed across peninsular India using headspace-solid phase microextraction-GC-flame ionization detection (HS-SPME-GC-FID). They found that these varieties contained 19 shared volatile compounds, which constituted more than 85% of the total volatile compounds; these volatile compounds had a typically pandan-like, green, and bean aroma [7]. Therefore, previous analyses of pandan leaves performed using different analytical methods have yielded volatile compounds that vary considerably in type, quantity, and aromatic structure. To better understand the effects of different processing methods on the volatile aroma–compound composition and the overall flavor of pandan leaves, a systematic and comprehensive study of the available flavor detection methods, processes, and statistical analysis methods must be conducted to provide a theoretical basis for further pandan-leaf processing and application.

HS-SPME-GC-MS enables accurate qualitative and quantitative analyses of different volatile compounds; however, this technique provides no sensory information. Olfactometric detection systems, which are electronic instruments that use the human nose to detect the odor intensity of analytes, can validate vague, non-subjective sensations using MS and matching to NIST libraries. In this way, samples can be correlated with all aroma types and complex analytical methods can be simplified to characterize and differentiate food products with distinctive aroma features.

Headspace gas chromatography–ion mobility spectrometry (HS-GC-IMS) is an effective analytical method for the separation and identification of trace volatile organic compounds (VOCs) in complex matrices [15]. The combination of GC and IMS offers enhanced separation capabilities and rapid responses. Therefore, HS-GC-IMS has a fast response, high analytical efficiency, and a wide detection threshold while also being easy to operate. This technique characterizes chemical ionic substances based on differences in the gas-phase mobilities of different ions in an electric field. HS-GC-IMS has been successfully applied to the analysis of honey [16] and coffee [17], which have complex flavor profiles and characteristic aromas, with easily overlooked flavor compounds being resolved. This method can detect volatiles with concentration resolutions as low as micrograms per liter, without the need for complex sample pre-treatment. Additionally, HS-GC-IMS can precisely identify volatiles in a non-targeted manner based on two-dimensional (2D) information in the GC retention time (RT) and IMS drift time. Currently, this technology is being applied to food additives [18], plants [19], tobacco [20], environment sensing [21], and medicine [22].

Principal component analysis (PCA) and orthogonal projections to latent structures discriminant analysis (OPLS-DA) are powerful statistical modeling tools that provide insights into separations between experimental groups based on high-dimensional spectral measurements from NMR, MS, or other analytical techniques. To prove that the ingredients tested are real and valid, multiple tests and multivariate statistical methods must be used to validate a method. This study represents the first investigation of the differences in volatile aroma components of fresh and dried pandan leaves through the aforementioned methods. Fresh pandan leaves were sourced from Hainan, China, with some samples subjected to sun- and freeze-drying treatments. The volatile aroma components of fresh, sun-dried, and freeze-dried samples were extracted and enriched using HS-SPME combined with GC-MS and GC-IMS, and were identified and classified using olfactory tests and OAV analytical techniques.

## 2. Materials and Methods

### 2.1. Materials

#### 2.1.1. Raw Materials

Fresh pandan leaves were collected in September 2022 from a pandan farm in Wanquan Colorful Science and Technology Agriculture Co., Ltd., (Wanning, China). The selected variety is representative and widely grown in Southeast Asia. To characterize the pandan-leaf aroma as accurately as possible, samples were randomly taken over 2 d of storage at 4 °C. In total, 2 kg of pandan leaves were extracted and processed without mechanical damage.

Fresh pandan leaves were artificially ground under liquid nitrogen drying and sieved through a 60-mesh sieve to obtain a powder. In the sun-drying process, the pandan leaves were naturally dried at 25 °C for 7 d, before being crushed through a 60-mesh sieve to obtain a powder. With reference to the optimization process [23], in the freeze-drying process, the pandan leaves were crushed, immersed in a water bath, centrifuged, vacuum freeze-dried, and cryogenically milled through a 60-mesh sieve to obtain powder samples. In this paper T1-1, T1-2, and T1-3 were used to name the fresh sun-dried and freeze-dried samples of pandan leaves, respectively. All prepared samples were stored in an ultra-low-temperature refrigerator at −80 °C until analysis prior to loading. The experimental analyses were performed in three batches.

#### 2.1.2. Chemicals

A standard mixture of n-alkanes (C5–C20), 2-octanol, 2-nonanone (as an internal standard, 99%), used to calculate KIs, and 2-AP (as an internal standard, 97%) was purchased from Energy-Chemical Reagents, Ltd. (Shanghai, China). A mixture of standards for the detection of six ketones (2-butanone, 2-pentanone, 2-hexanone, 2-heptanone, 2-octanone, and 2-nonanone) was purchased from Aladdin Chemical Reagent Co. (Shanghai, China). Chromatography-grade ethanol was purchased from Sigma Chemical Reagents (St. Louis, MO, USA).

### 2.2. HS-GC-IMS Preparations

Each sample was transferred to liquid nitrogen for rapid pre-cooling, milled and sieved through a 60-mesh sieve, packed in a vacuum-sealed food-grade polyethylene plastic storage bag, and stored frozen at −80 °C. For testing, 1.000 g of each sample was accurately weighed on an electronic balance (AL104-1C Electronic Analytical Balance, Mettler Toledo, Shanghai, China) and mixed with 1000 μL of the internal standard (10 mg/L of 2-nonanone in ethanol solvent) before being placed in a 20 mL HS injection bottle. The sample was then incubated at 60 °C and shaken at 500 r/min for 15 min. For each sample, measurements were performed for three parallel groups, and an empty sample bottle was employed as blank control.

### 2.3. HS–GC–IMS Analysis

The samples were analyzed using an Agilent 8890 gas chromatograph (G.A.S., Dortmund, Germany) equipped with a DB-5MS capillary column (30 m × 0.25 mm, 0.25 μm) and an IMS system with a CTC-PAL 3 static HS autosampling unit (CTC Analytics AG, Zwingen, Switzerland) and a feeding needle (Dortmund, Germany). For the initial column, the GC oven temperature was held at 40 °C for 4 min, before being increased to 100 °C at a rate of 6 °C/min. This was followed by another increase to 250 °C at a rate of 4 °C/min and, finally, holding for 5 min. The GC carrier gas was high-purity helium (≥99.999%), which was supplied at a 1.5 mL/min flow rate in constant flow mode, while the GC oven maintained an inlet temperature of 250 °C. No injection splitting was observed.

The IMS was set to splitless mode, the column temperature was set to 60 °C, and the IMS temperature was 45 °C. High-purity nitrogen (≥99.99%) was used as the drift gas, with a 75 mL/min flow rate. B-rays (3H, 300 MBq) were used as the ionization source, in positive ion mode and with a 53 mm migration tube length. The RIs and drift times were compared with those in the G.A.S. GC-IMS library (Dortmund, Germany) to enable volatile compound identification. The RIs were calculated based on the C4–C9 ortho-ketones, for a 500 V/cm electric field strength and a 45 °C migration tube temperature.

Mixed standards of six ketones were detected and RT and retention index (RI) calibration curves were established. The substance RIs were then calculated from the target RTs. The target volatiles were analyzed qualitatively and semi-quantitatively using the GC RI database (NIST 2020) incorporated in the VOCal software package (version 4).

### 2.4. HS–SPME

For most volatile compounds, a 10–15 min equilibration time is sufficient [7]. Therefore, a 10 min equilibration time was selected for this study. With reference to the conditions and results of the SPME optimization test reported by Wakte, a 10 mm 80 μm divinylbenzene/carboxen/polydimethylsiloxane fiber assembly (Agilent, Santa Clara, CA, USA) was selected for the sample analysis. Based on our pre-optimized extraction conditions, the samples were accurately weighed at 1.000 g each on a balance (AL104-1C Electronic Analytical Balance, Mettler Toledo Instruments, Geneva, Switzerland) and placed into 20.00 mL HS injection vials spiked with 5 μL of the internal standard (1.02 mg/mL of 2-octanol in ethanol solvent). The vials were then immediately sealed and equilibrated for 10 min at 40 °C. The samples were then analyzed at an extraction temperature of 40 °C and an extraction time of 20 min. The fibers were placed into the inlet port for 5 min using manual HS injection with an SPME injector; they were then thermally desorbed at 250 °C.

### 2.5. GC–O–MS Analysis

The SPME fibers were thermally desorbed for 5 min on an 8890GC-5977MS GC-MS coupler (Agilent, USA) with an HP-5MS capillary column (30 m × 0.25 mm, 0.25 μm). The GC effluent was separated at a 1:1 ratio at the MS detector and the sniffer port for analysis. The carrier gas was high-purity helium (≥99.999%) at a constant flow rate of 1.5 mL/min. The GC temperature was initially maintained at 40 °C for 4 min. This temperature was then increased to 100 °C at a rate of 6 °C/min for 5 min, held for 5 min, increased to 250 °C at a rate of 4 °C/min and, finally, held for 5 min. The injector, MS-interface, and ion-source temperatures were held at 250, 280, and 230 °C, respectively. The electronic ionization was set to 70 eV with a m/z range of 50–550.

### 2.6. Aroma Extraction Dilution Analysis

An aroma extraction dilution analysis (AEDA) dilution test was performed under the gas chromatography–olfactometry–mass spectrometry (GC-O-MS) conditions of Section 2.5. An olfactometer (ODP4, GERSTEL) was used for olfactory analysis of the GC effluent substances, with the transfer temperature between the gas chromatograph and olfactory detector set to 280 °C. Three evaluators with the best olfactory statuses were selected from ten panelists with more than three years of experience and a familiarity with food volatiles. Based on the previous reports [24,25], an AEDA dilution test was performed via GC-O-MS on the T1-1, T1-2, and T1-3 samples. The samples were diluted stepwise with dichloromethane at a 1:2 ratio to obtain extract dilution ratios of 1:1, 1:2, 1:4, etc., up to the highest value of 1:512. Each dilution was analyzed using GC-O on a HP-5MS column, and three experienced sensory panel members evaluated each dilution using GC-O. During sniffing, the evaluators were required to describe the sensory characteristics of the corresponding substance and record the RT and aroma type until no aroma was detected. Each member repeated each experiment thrice. The flavor dilution (FD) factor for each compound was defined as the highest dilution that could be smelled. The compounds identified for the three samples (T1-1, T1-2, and T1-3) were validated against the standards in this test.

### 2.7. Qualitative and Relative Quantitative Analysis of Aroma Components

#### 2.7.1. Qualitative Analysis

C5–C20 n-alkanes were analyzed under the conditions described in Section 2.5, their RT values were recorded. The RIs of the volatiles were calculated according to the linear retention index referring to the IUPAC Compendium of Chemical Technology [26]:

(1) $$RI=100\times \left(n+\frac{T_{Y}-T_{n}}{T_{n+1}-T_{n}}\right)$$

where n and n + 1 represent the number of carbon atoms in the n-alkanes before and after the target compound Y elutes. It has been observed that T_n_ < T_Y_ < T_n+1_. Generally, the number of carbon atoms, n, in n-alkanes is greater than 4.

#### 2.7.2. Relative Quantitative Analysis

Referring to the internal standard calculation method of report [27], the relative content of any volatile compound Y in the sample was determined as follows:

(2) $$C_{Y}={\mathrm{f\prime}}_{Y}\times \frac{A_{Y}}{A_{X}\times m_{Y}}\times m_{X}$$

where A_X_ and C_X_ are the peak area and relative concentration of the internal standard X, respectively; A_Y_ and m_Y_ are the peak area and relative concentration of compound Y; and f′_Y_ is the relative mass correction factor of compound Y with respect to the internal standard. In this experiment, the relative correction factor for each compound was 1.000.

The volatiles were determined by comparing the mass spectrum of each analyte to the NIST 17 standard library. The actual RI of compound Y was then calculated and compared with the theoretical value (RI’). In addition, the odor compounds were reconfirmed according to the Flavor-Base FEMA-reported standard odor description database.

### 2.8. Odor Activity Value Calculation

With reference to the method used in report [28], the ratio of the concentration of an olfactory substance *C* to its perception threshold *T* was defined as the OAV. The OAV can be used to determine whether a compound is a significant contributor to the aroma [29]. For any component *Y*,

(3) $${OAV}_{Y}=\frac{C}{T}$$

where C is the relative content of each volatile compound (%), and T is the corresponding water (fresh) or cellulose (dried) threshold (mg/kg), respectively. An OAV > 1 indicates that the substance component may have a direct impact on the overall odor. Within a certain range, a larger OAV indicates a greater contribution of the substance to the overall odor.

### 2.9. Data Processing and Statistical Analysis

The GC-O-MS data were analyzed using MassHunter qualitative software version F.01.03, the raw data were maintained using Microsoft Excel 2019, and figures were drawn using OriginPro (version 2022, USA), SIMCA (version 14.1, Sweden), and TBtools (version 1.1, China). The IMS data were processed using Xcalibur Software (version 4.3, Germany), with Reporter and Gallery Plot being used for flavor analysis. VOCal data processing software (version 0.4.03, Germany) was used to obtain 3D, 2D, difference, and fingerprint plots of the volatile constituents for the comparison of the VOCs within the different samples.

All tabular data analysis was conducted using SPSS 13.0. Analysis of variance (ANOVA) tests were performed in triplicate using mean ± standard deviation values. Significant differences were verified via ANOVA and the Duncan multiple range test (*p* < 0.05). PCA and OPLS-DA plots, along with validation models, were obtained using SIMCA.

## 3. Results

### 3.1. HS-GC-IMS Analysis

Figure S1 shows the 3D HS-GC-IMS spectra for the three pandan-leaf treatment samples considered in this study. In each spectrum, the three axes represent the migration time (*X* axis), RT (*Y* axis), and signal peak intensity (*Z* axis). To further visualize and compare the differences in the volatile compound compositions, the spectrum of the T1-1 sample was selected as a reference and subtracted from the spectra of the other samples.

**Figure 1:**
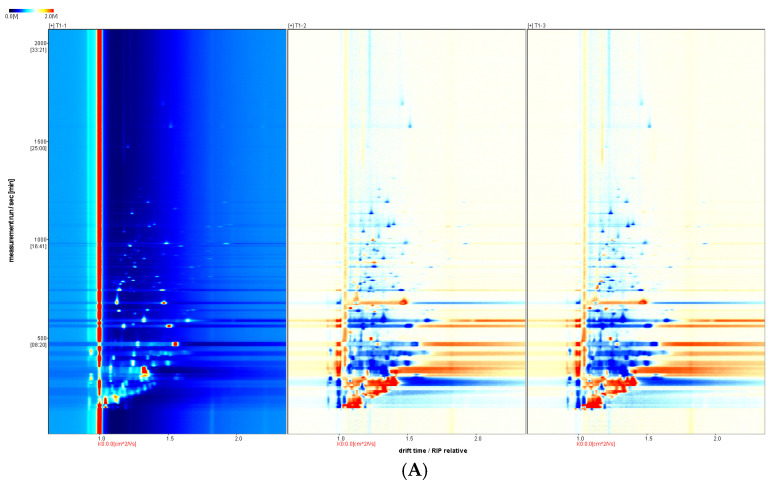

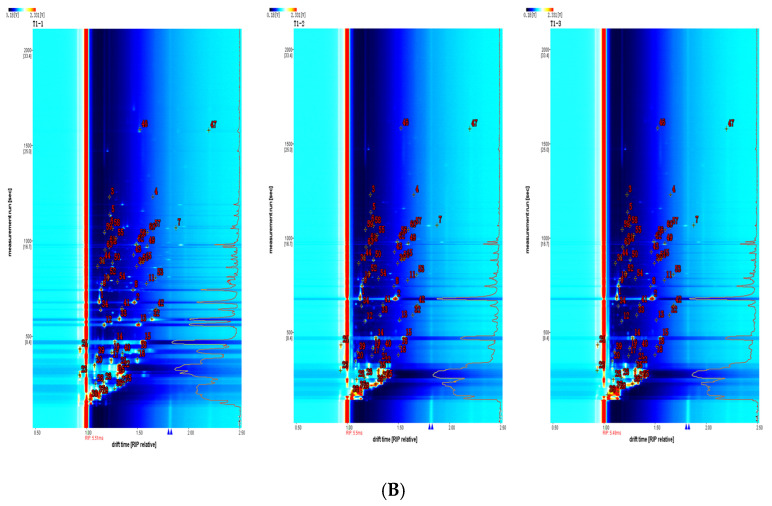
Comparison of volatiles from different samples by HS-GC-IMS. (**A**) Two-dimensional GC-IMS difference spectrum of volatile components in pandan leaves. (**B**) Qualitative GC-IMS spectra of volatile components in pandan leaves.

For ease of observation, a top-view comparison is shown in Figure 1A. The abscissa in the 2D GC-IMS spectrum represents the Reactant Ion Peak (RIP) and the ordinate represents the GC RT (s). Each point on either side of a RIP represents a VOC, and two or more spots may represent different aggregates of the same organic matter, such as dimers or multimers, which were formed during GC-IMS analyses. The odor-component content is indicated by the change in the substance peak intensity. Here, the peak intensity of the volatile substance is represented using color shading from blue to red; the darker the color, the greater the peak intensity and the higher the substance content. The figure has shown that the VOCs of the different treatments could be separated via GC within 30 min, and that the components were mainly distributed between 0 and 1500 s, with a drift time of 1.0 ms to 1.5 ms. The three treatments seem quite different; moreover, the 3D peaks were available for comparison (Figure S1).

**Figure 2:**
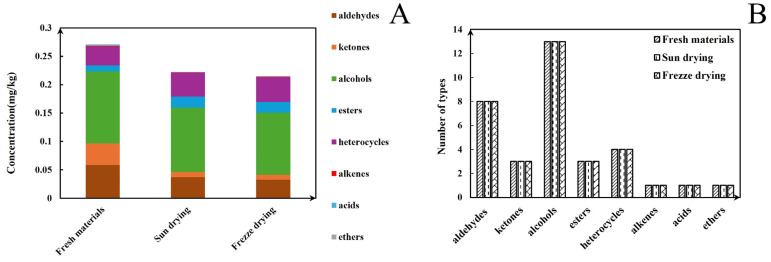
(**A**) Contents of different types of volatiles detected under HS-GC-IMS in different samples. (**B**) Volatile types in pandan leaves in different samples.

The GC-IMS analysis results are presented in Table 1. The VOCal plug-in was used to characterize the volatile components in each sample; the characterization spectra are shown in Figure 1B. A total of 63 signal peaks were determined for the three samples. The results matched the GC × IMS database, with 58 components being accurately characterized. Among the detected components, 24 pairs of components appear as a monomer (“M”) or dimer (“D”) because of the ionization of the protonated neutral component before passing through the drift tube during the detection process (see the Supporting Information for more details). A total of 34 compounds were identified using HS-GC-IMS, with a rich composition of VOCs, including 2 nitrogenous compounds, 14 alcohols, 8 aldehydes, 4 ketones, 2 esters, 1 oxygenated compound, 1 alkene, 1 acid, and 1 ether. The volatile-component breakdown is listed in Table 1. In terms of the total volatile concentration (Figure 2), the order was as follows: T1-1 > T1-2 > T1-3.

**Figure 3:**
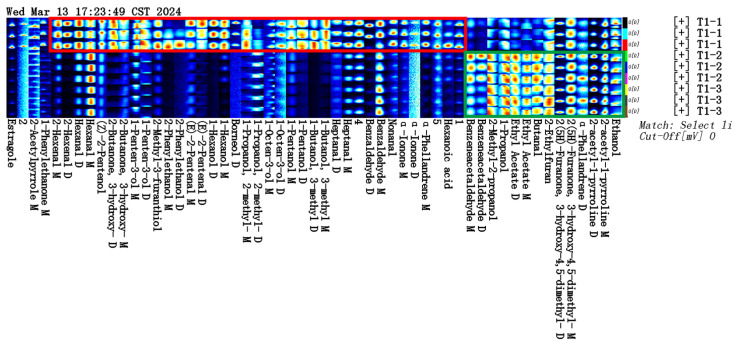
Aroma fingerprint based on volatile component contents determined by HS-GC-IMS.

To further determine and compare the volatile substances obtained under the different processing methods, the fingerprints of all the volatile substances were established (Figure 3). The aroma compound compositions and distributions differed greatly between the treatments. As shown in the red box in Figure 3, borneol, hexenal, (Z)-2-pentenal, 3-hydroxy-2-butanone, 1-penten-3-ol, 2-methyl-3-furylthiol, benzaldehyde, pentanol, (E)-2-pentenal, heptanal, hexanol, nonanal, 2-phenylethanol, and α-ionone had high concentrations in T1-1. In contrast, 2-ethylfuran, 2-methylpropanol, ethyl acetate, 2-acetylpyrroline, ethanol, butanal, phenylacetaldehyde, and α-phellandrene were more abundant in T1-2 and T1-3, as indicated by the green box.

### 3.2. HS-SPME-GC-O-MS Analysis of Volatile Compound Profiles

**Figure 4:**
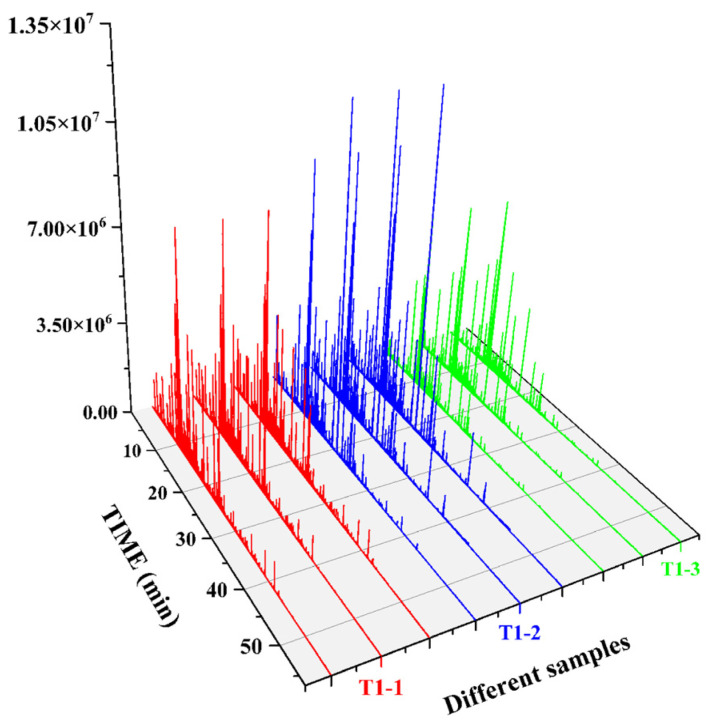
GC-O-MS total ion chromatograms of three samples.

**Figure 5:**
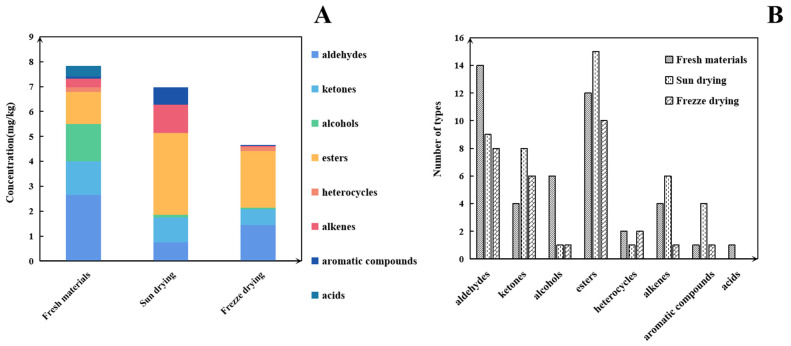
(**A**) Content of different types of volatiles detected under HS-SPME-GC-O-MS in different samples. (**B**) Volatile types in pandan leaves in different samples.

Based on our pre-optimized HS-SPME-GC-MS sample preparation conditions, the total ion chromatograms of the three samples are shown in Figure 4. The volatile compounds identified through this analysis are listed in Table 2. As shown in Figure 5A, and Table 2, the volatile compound fractions of the pandan leaves were varied, with 72 compounds being identified and classified into eight major groups: 19 aldehydes, 11 ketones, 6 alcohols, 3 heterocyclic groups, 4 aromatic compounds, 20 esters, 8 alkenes, and 1 acid. Figure 5B shows the types and quantities of volatile compounds produced in the employed processes, which mainly consisted of esters, aldehydes, and ketones. Among these, 45, 44, and 29 VOCs were identified for the fresh, sun-dried, and freeze-dried pandan leaves, respectively. Notably, the ester constituents were more abundant in T1-2 and T1-3. In principle, alcohol acyltransferases and dehydrogenases play key roles in the biosynthesis of esters, amino acids, sugars, and lipids, which must be converted to acids, aldehydes, or alcohols before being converted to esters [30]. Acids and alcohols can be converted directly by alcohol acyltransferases, whereas aldehydes must be converted to acids or alcohols before being converted to esters [31]; this aspect is coincidentally illustrated by the greater abundance of aldehydes in T1-1. As an example of the reaction pathways of the substances examined in our study, hexanal can be generated from linoleic acid by the actions of lipoxygenase and hydroperoxide lyase, followed by the oxidation of hexanal to hexanoic acid or the generation of hexanol by the action of alcohol dehydrogenase. Further esterification can generate esters such as ethyl hexanoate [32].

The total volatile matter content was in the order of fresh > sun-dried > freeze-dried. This order is consistent with the HS-GC-IMS results and may be related to the aromatic-substance formation or volatilization caused by the drying process. During the different pretreatment processes, these VOCs may undergo changes before being frozen that are affected by some biochemical metabolic activities; therefore, the formation of aroma during drying is a dynamic process [33]. When it comes to sun-drying, most of the basic and non-basic components of food are degraded to some extent, yielding a wide variety of odorous compounds. Volatile compounds are usually released from fresh product tissues before drying, when cell fragmentation occurs and enzymes and organic substrates isolated from different cell compartments interact. At this stage, the various fatty acids, amino acids, and carbohydrates in the food act as aroma precursor substances, which could be converted to form different volatile compounds under the action of key enzymes. A variety of volatiles collaborate to form an aroma that is unique to the sun-drying method [34].

In the vacuum freeze-drying process, alcohols may oxidize into corresponding aldehydes, ketones, and acids, for example, 1-hexen-3-ol can be converted into 2-hexenal and 2-hexenoic acid. Secondly, the dehydration caused by drying halts many enzymatic reactions in the Pandan leaves. Upon rehydration, water molecules serve as a medium for various enzymatic reactions. The significant decreased content of compounds such as 1-octen-3-ol and 2-octanone after drying can likely be attributed to these enzymatic reactions. Lastly, the diversity of isoenzymes in Pandan leaves may also contribute to different outcomes, although isoenzymes catalyze the same chemical reactions, and their physicochemical properties and molecular structures can differ, leading to varying results [35].

**Figure 6:**
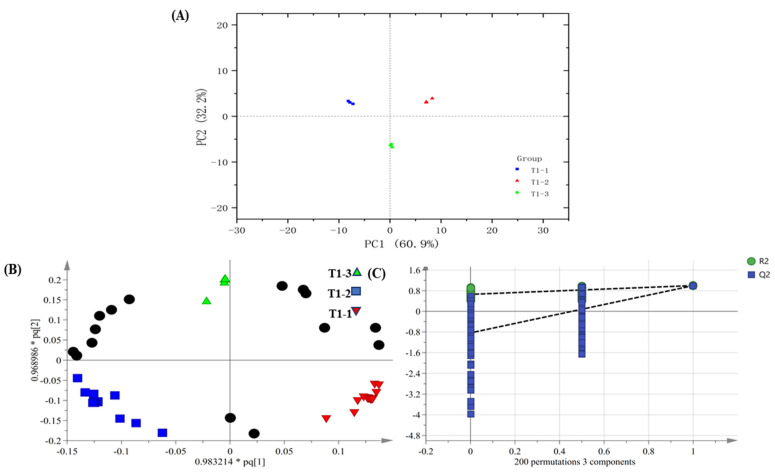
(**A**) PCA score diagram, (**B**) OPLS-DA (The black circle, along with other shapes, represents the significance of compounds), and (**C**) model cross-validation results of pandan leaves components based on HS-SPME-GC-O-MS data (The black line represents a curve trend with mathematical significance).

PCA shows the separation among the differently processed samples (Figure 6A). These results have indicated compositional differences in the pandan-leaf volatile components subjected to the different processes. To verify the significance of the aroma differences and to differentiate among the treatments in Table 2, OPLS-DA was employed by taking 72 shared aroma components as dependent variables and the treatments as independent variables (Figure 6B). The fit indexes for the independent and dependent variables (R2x and R2y, respectively) were 0.931 and 0.996, respectively. The model prediction index (Q2) was 0.992, and R2 and Q2 values exceeding 0.5 were obtained, indicating acceptable model fitting [36]. After 200 substitution tests, as shown in Figure 6C, the intersection of the Q2 regression line with the vertical axis was less than 0, which also indicated that there was no model overfitting and that the model validation was effective. These results are considered useful for the future identification of pandan constituents in terms of their processing methods.

**Figure 7:**
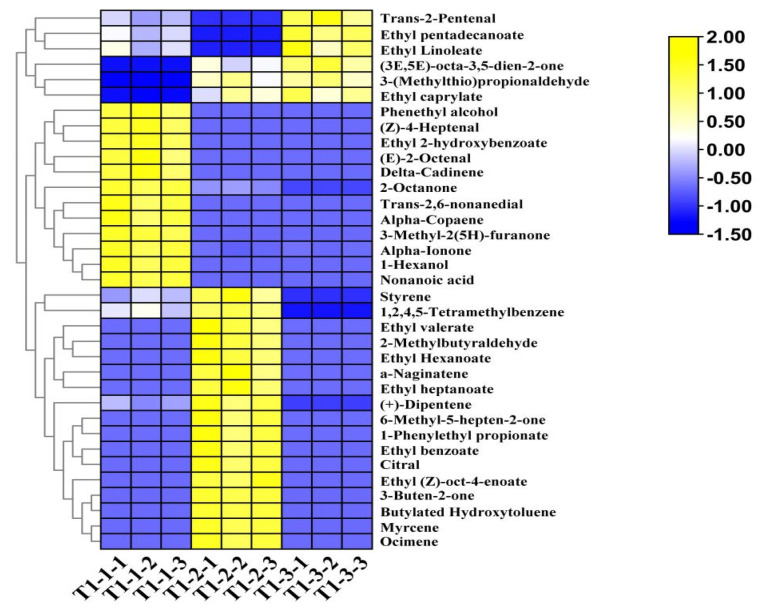
Differential aroma component clustering heat map of pandan-leaf components based on HS-SPME-GC-O-MS data.

The total ion flow chromatogram (Figure 6C) was determined using the peak RT and mass-spectrometry signal intensity parameters of the aroma components. Obvious differences existed between the ion spectra of the different samples. Regarding the differences between the fresh samples and those treated by the different drying processes, some of the aroma components were similar, but the contents of each substance type differed. To further clarify the affinity between the volatile flavor profiles of the different samples, 35 different aroma substances were screened out from the total volatile substances according to the following criteria: *p* < 0.05 and VIP > 1. A hierarchical clustering heat map was drawn based on the substance content data (Figure 7), from which 10 esters, 7 aldehydes, 6 alkenes, 5 ketones, 2 alcohols, 2 aromatic compounds, 2 heterocyclic compounds, and 1 acid were selected. Region A, highlighted in yellow, contains 12 different aromatic constituents of the fresh pandan-leaf samples, including phenethyl alcohol, (2E,6E)-nona-2,6-dienal, nonanoic acid, and α-ionone. Region B contains the 17 most prominent differential aromatic components in the sun-dried sample, including citral, styrene, 3-buten-2-one, 1-phenylethyl propionate, and ethyl hexanoate. Region C shows the six aroma components with prominent contents in the freeze-dried sample, namely, trans-2-pentenal, 3-(methylthio) propionaldehyde, (3E,5E)-octa-3,5-dien-2-one, ethyl caprylate, ethyl pentadecanoate, and ethyl linoleate.

### 3.3. Key AEDA-Identified Aroma-Active Compounds

Volatiles from the SPME extracts were analyzed using the AEDA sniffing dilution test to determine the volatile compound contributions to the overall aromas of the pandan leaves subjected to different processes. An AEDA sniffing test for 72 compounds was performed based on the stationary-phase RIs, compound-standard RTs, and gradient dilutions utilizing the above GC-O-MS conditions. With the GC-O-MS method, aroma-active compounds were determined based on the air matrix, which differs from the dichloromethane solvent matrix used in the sniff test. Therefore, this work combined the substance content with the odor perception threshold, defined as the OAV, to statistically analyze the validation test results (see Table S1 of the Supporting information for details).

### 3.4. OAVs of Volatile Compounds in Three Pandanus amaryllifolius Roxb. Samples

**Figure 8:**
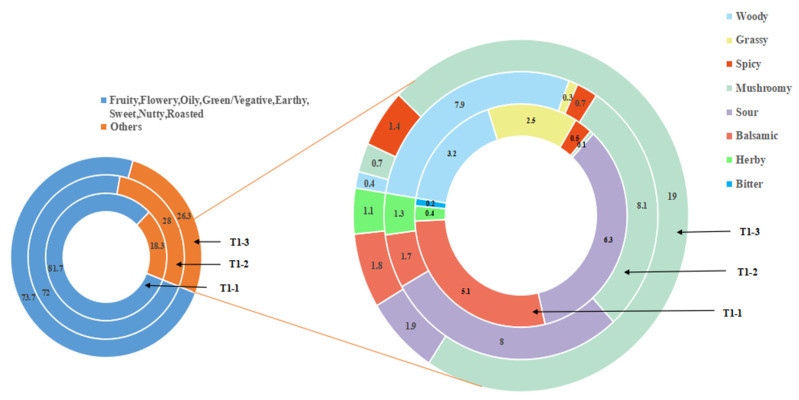
Flavor wheel of the aroma compounds present in pandan leaves under different processes based on OAVs.

The aroma component content is not sufficient for determining the aroma profile. The odor activity value (OAV) is a common method to objectively describe the contribution value of aroma components to the aroma style of a sample [37]. Herein, this work calculated the OAVs of the volatile compounds in all three samples based on the relative substance concentration and detection threshold. Compounds with an OAV > 1 were defined as key odorants (the odorants had OAVs ≥ 1). Table 3 lists 43 volatile compounds with an OAV > 1 and their corresponding dilution factors, grouping the compounds based on their odor properties. Finally, this work plotted the flavor wheels of the different pandan-leaf samples based on the OAVs of the odor properties (Figure 8).

The experimental results (Table 3) also showed that the fresh, sun-dried, and freeze-dried samples had 31, 28, and 18 key odorants, respectively. Further, there were 13 common components among the sample types. This work believes that these 13 compounds together constitute the volatile aroma of pandan leaves, which is an important underlying flavor. Five of the components were aldehydes: hexanal (OAV = 33.86 ± 8.29–73.71 ± 3.64), nonanal (114.85 ± 24.5–203.25 ± 67.55), phenylacetaldehyde (66.25 ± 3.58–85.25 ± 4.33), β-cyclocitral (4.27 ± 0.36–12.63 ± 0.96), and butanal (2.80 ± 0.04–7.84 ± 0.15); four were esters: ethyl caprylate (3.06 ± 0.48–37.83 ± 6.74), ethyl nonanoate (6.78 ± 0.84–21.92 ± 4.99), ethyl caprate (87.25 ± 10.25–712 ± 245.75), and ethyl laurate (106.85 ± 30.45–205.55 ± 78.35); three were ketones: 3-hydroxy-2-butanone (0.44 ± 0.01–2.43 ± 0.08), acetophenone (5.18 ± 0.7–27.44 ± 5.2), and α-ionone (22.6 ± 2.33–124.53 ± 6.98); and one was a nitrogenous compound: 2-AP. Indeed, 2-AP exhibited the highest OAV among the shared compounds (718.01 ± 27.28–1080.06 ± 13.22) and, for all three treatments, 2-AP had OAVs of 700–1000 with FDs of 256–512. In conclusion, 2-AP was the most important odor-active compound in the fresh pandan leaves considered in this study, and the main source of aroma in the processed pandan leaves. Its olfactory aroma types were roasted and sweet.

T1-1 had 31 aroma components with an OAV > 1, which constituted the basic (13) and characteristic (18) key odorants. This work concluded that there were 18 volatile components that could be taken as characteristic aromatic substances, as listed in Table 3. Among them, nonanoic acid, butyl acrylate, decyl aldehyde, (2E,6E)-nona-2,6-dienal, and other aroma components with an OAV > 50 were identified; these components can contribute to green, floral, fruity, and cheesy aromas with high sensory scores. In particular, a higher OAV was obtained for (2E,6E)-nona-2,6-dienal, which was a fresh and delicate fragrance, in T1-1 than in the other samples. This component may enhance the aroma that is characteristic to fresh pandan leaves. For T1-2, 28 aroma components with an OAV > 1 were determined; these components were considered to constitute the basic (13) and characteristic (15) key odorants. Among them, 3-buten-2-one (flowery, woody) and ethyl undecanoate (fruity, coconut) had OAVs of almost 1000, and a sensory predominance of fruity and woody aromas was determined. Methyl benzoate (flowery, fruity, green, herby), ethyl valerate (fruity, herby), and isovalerylaldehyde (fruity, sweet) had an OAV > 50, indicating that these components may also contribute significantly to the aromatic characteristics of sun-dried pandan-leaf samples. Further, the most significant differences in the OAVs among the three samples were obtained for these five substances; thus, they may be the main aromatic components differentiating the different treatments. For T1-3, 18 aroma components had an OAV > 1 and were considered to constitute the basic (13) and characteristic (5) key odorants of the freeze-dried samples.

The OAVs of most aromatic components of the volatile components were similar. For example, ethyl undecanoate (fruity, coconut) and 3-(methylthio) propionaldehyde (roasted, mushroomy) appeared in T1-2; these are new products discovered after processing and drying. Benzaldehyde (roasted, nutty, almond) and trans-2-hexenal (juicy, green) were found in T1-1. However, the only compound that distinguished the freeze-dried sample from the fresh and sun-dried samples was a volatile compound found in the former; namely, isophorone (green, herby), which has a camphor odor characteristic that made the freeze-dried sample easier to distinguish than the samples of the other two treatments. The differential aroma substance of interest was 3-(methylthio) propionaldehyde, which was present in both the processed sun- and freeze-dried samples, with an OAV > 1000. It has a roasted aroma and mushroomy flavor that may not be obvious but that constitutes the base flavor of the volatile aroma. This flavor may be related to the short heating time of the processed powder. Because of the special nature of sun-drying technology, the integrity and activity of the pandan-leaf cells are maintained for a long time during processing, allowing for sufficient enzymatic reactions. During processing, the leaves are subjected to various stresses and, particularly, a comprehensive stress response during the greening process. This stress response is related to dehydration reactions, sustained temperature-induced mechanical damage during sun-drying, and the Maillard reaction [38].

In addition, most of the key odorants had high FD values, indicating consistency between the calculated OAVs of these compounds and the AEDA results; however, exceptions exist. For example, ethyl pentadecanoate had an FD value of 16 in both T1-1 and T1-3, whereas its OAVs were less than 1. These results indicate interactions between the various volatile substances, rather than simple additive relationships, which may affect the retention or release of flavor compounds. These results also confirm that the combination of smell and instrumentation plays a crucial role in food flavor research.

Although this study could investigate volatile aromas in terms of the recombination of aroma substances to discuss the reproduction of artificially blended aromas, just as flavor is the result of a multitude of factor-driven outcomes, this work may be able to discuss more profound flavor mechanisms in terms of metabolites and genes of samples from different regions or varieties.

### 3.5. Comparative Analysis of HS-SPME-GC-O-MS and HS-GC-IMS Results

In this study, a total of 93 VOCs were identified in three pandan-leaf samples utilizing HS-GC-IMS and HS-SPME-GC-MS. In addition to these, 13 VOCs (benzaldehyde, 2-hexenal, hexanal, 2-acetylpyrrole, 1-hexanol, 2-ethylfuran, (E)-2-pentenal, nonanal, acetophenone, α-ionone) were detected by both analytical methods. Notably, compounds such as estragole, geraniol, 2-butanone, and 2-Methyl-3-furanthiol were observed solely via GC-IMS, suggesting that these compounds may have a negligible contribution to the aromatic profile of pandan leaves. Furthermore, the contribution of VOCs was evaluated using the OAV methodology. The analysis identified 43 compounds with an OAV > 1, and 13 of these compounds were confirmed by both GC-IMS and SPME-GC-O-MS. These include 2-AP, hexanal, nonanal, phenylacetaldehyde, β-cyclocitral, butanal, ethyl caprylate, ethyl nonanoate, ethyl caprate, ethyl laurate, 3-hydroxy-2-butanone, acetophenone, and α-ionone, all of which are hypothesized to significantly influence the characteristic aroma associated with “glutinous rice” or pandan flavor. GC-O-MS demonstrates a higher capacity for identifying volatile compounds compared to GC-IMS, and the concentrations of VOCs discovered by GC-IMS are lower than those identified by GC-O-MS. Consequently, the integration of GC-IMS and GC-O-MS methodologies is proposed to enhance the comprehensive identification and quantification of aroma compounds in complex matrices.

## 4. Conclusions

All in all, 72 and 34 volatile compounds of fresh pandan and its processed leaves were successfully identified using HS-SPME-GC-O-MS and HS-GC-IMS, respectively. Differential metabolites were analyzed under the criteria of a VIP > 1 and *p* < 0.05, and the volatile compounds were qualitatively analyzed using an AEDA dilution olfactory test. The differences between fresh leaves and processed leaves were assessed through comparative olfactory tests and OAV results, demonstrating that pandan-leaf aroma can be classified into 16 aroma types. The comparative analysis of the GC-IMS and SPME-GC-O-MS results yielded 13 consensus volatiles, including 2-AP, hexanal, nonanal, phenylacetaldehyde, β-cyclocitral, butanal, ethyl caprylate, ethyl nonanoate, ethyl caprate, ethyl laurate, 3-hydroxy-2-butanone, acetophenone, and α-ionone, which are the key components contributing to the characteristic “glutinous rice” flavor of pandan leaves. Furthermore, there were 31, 28, and 18 compounds in the fresh, sun-dried, and freeze-dried samples, respectively. The differences in these compounds result in subtle variations in the aroma of the various states of pandan leaves. This study provides a theoretical basis for further research on key substances related to the characteristic flavors of plants and offers data supporting the selection of pandan-leaf processing styles and aroma recombination practices.

## Figures and Tables

**Table 1:** Contents of volatile components in samples identified by HS-GC-IMS.

No.	Compound	RI ^1^	RT [s]	RI’ ^1^	Dt [a.u.]	Perception Threshold (mg/kg) ^2^	Relative Concentration (µg/kg) ^3^		
							T1-1	T1-2	T1-3
1	2-acetyl-1-pyrroline M ^4^	932	688.80	923	1.12	0.00003	11.19 ± 0.21 ^b^	13.32 ± 0.18 ^a^	12.84 ± 0.09 ^a^
2	2-acetyl-1-pyrroline D ^4^	923	675.90	923	1.47	0.00003	10.35 ± 0.61 ^b^	18.64 ± 0.36 ^a^	19.56 ± 0.30 ^a^
3	Geraniol M	1220	1230.48	1221	1.23	0.049	1.05 ± 0.06 ^a^	0.77 ± 0.02 ^b^	0.80 ± 0.04 ^a^
4	Geraniol D	1220	1228.56	1221	1.65	0.049	1.12 ± 0.05 ^a^	1.10 ± 0.04 ^a^	1.05 ± 0.03 ^a^
5	Estragole	1180	1136.37	1181	1.23	na	2.15 ± 0.06 ^a^	0.81 ± 0.06 ^b^	0.74 ± 0.01 ^c^
6	Borneol M	1151	1074.91	1150	1.22	0.052	0.87 ± 0.04 ^a^	0.62 ± 0.05 ^b^	0.53 ± 0.03 ^b^
7	Borneol D	1148	1067.23	1150	1.88	0.052	0.38 ± 0.02 ^a^	0.21 ± 0.01 ^b^	0.22 ± 0.01 ^b^
8	Benzaldehyde D	967	743.68	964	1.46	0.085	2.52 ± 0.84 ^a^	0.87 ± 0.02 ^b^	1.04 ± 0.01 ^b^
9	Benzaldehyde M	968	747.07	964	1.15	0.085	3.95 ± 0.59 ^a^	3.59 ± 0.06 ^a^	4.48 ± 0.04 ^a^
10	1-Octen-3-ol M	986	775.80	985	1.15	0.005	2.27 ± 0.08 ^a^	1.77 ± 0.02 ^b^	2.20 ± 0.03 ^a^
11	1-Octen-3-ol D	986	775.80	985	1.59	0.005	0.39 ± 0.01 ^a^	0.28 ± 0.01 ^b^	0.28 ± 0.01 ^b^
12	2-Hexenal M	854	557.36	857	1.18	0.03	5.94 ± 0.09 ^a^	1.95 ± 0.10 ^b^	1.37 ± 0.03 ^c^
13	2-Hexenal D	858	564.71	857	1.51	0.03	10.98 ± 0.10 ^a^	0.97 ± 0.027 ^b^	0.69 ± 0.0056 ^c^
14	Hexanal M	802	471.19	802	1.28	0.0014	7.70 ± 0.03 ^b^	7.91 ± 0.02 ^a^	7.13 ± 0.04 ^c^
15	Hexanal D	802	470.42	802	1.56	0.0014	15.84 ± 0.36 ^a^	7.35 ± 0.16 ^b^	4.86 ± 0.15 ^c^
16	3-Methylbutanol D	747	378.79	744	1.50	0.125	8.67 ± 1.57 ^a^	0.64 ± 0.04 ^b^	0.71 ± 0.03 ^b^
17	3-Methylbutanol M	744	374.89	744	1.24	0.125	9.61 ± 0.25 ^a^	1.33 ± 0.01 ^b^	0.79 ± 0.01 ^c^
18	Pentanol D	773	423.63	771	1.52	0.153	3.18 ± 0.07 ^a^	0.47 ± 0.01 ^b^	0.37 ± 0.01 ^b^
19	Pentanol M	768	413.88	771	1.25	0.153	4.77 ± 0.19 ^a^	2.07 ± 0.04 ^b^	1.32 ± 0.01 ^c^
20	3-Hydroxy-2-Butanone M	726	347.59	720	1.08	0.014	5.66 ± 0.16 ^a^	2.50 ± 0.07 ^c^	2.93 ± 0.02 ^b^
21	3-Hydroxy-2-Butanone D	723	342.72	720	1.32	0.014	28.41 ± 1.03 ^a^	3.59 ± 0.07 ^b^	3.00 ± 0.08 ^b^
22	1-Penten-3-ol D	710	323.97	686	1.35	0.05	14.67 ± 0.88 ^a^	5.05 ± 0.06 ^b^	3.53 ± 0.07 ^c^
23	1-Penten-3-ol M	690	297.27	686	0.93	0.05	5.61 ± 0.26 ^a^	2.57 ± 0.04 ^c^	2.71 ± 0.03 ^c^
24	(*Z*)-2-Pentenol	779	434.12	783	0.94	0.72	5.10 ± 0.15 ^a^	1.99 ± 0.04 ^b^	1.45 ± 0.06 ^c^
25	2-Methylpropanol D	635	249.57	647	1.37	0.033	3.18 ± 0.71 ^b^	5.56 ± 0.13 ^a^	4.64 ± 0.09 ^a^
26	2-Methylpropanol M	647	259.18	647	1.17	0.033	7.20 ± 0.62 ^a^	2.10 ± 0.05 ^b^	2.06 ± 0.07 ^b^
27	Ethyl Acetate D	628	244.44	628	1.33	3.42	5.80 ± 0.32 ^b^	13.69 ± 0.22 ^a^	12.94 ± 0.43 ^a^
28	Ethyl Acetate M	635	250.21	628	1.09	3.42	1.75 ± 0.52 ^b^	2.82 ± 0.01 ^a^	2.89 ± 0.03 ^a^
29	2-Methyl-2-propanol	541	185.48	530	1.14	14	12.71 ± 0.79 ^c^	27.31 ± 0.47 ^b^	30.47 ± 0.23 ^a^
30	2-Acetylpyrrole D	1040	865.30	1037	1.49	2	0.26 ± 0.01 ^a^	0.27 ± 0.02 ^a^	0.27 ± 0.02 ^a^
31	2-Acetylpyrrole M	1041	866.94	1037	1.11	2	0.72 ± 0.01 ^a^	0.65 ± 0.03 ^ab^	0.60 ± 0.01 ^b^
32	Hexanol D	872	591.07	872	1.64	0.034	4.78 ± 0.18 ^a^	0.48 ± 0.02 ^b^	0.42 ± 0.01 ^b^
33	Hexanol M	872	591.07	872	1.32	0.034	7.11 ± 0.09 ^a^	1.37 ± 0.05 ^b^	0.43 ± 0.01 ^c^
34	2-Methyl-3-furanthiol	898	638.68	890	1.14	0.007	3.16 ± 0.16 ^a^	0.90 ± 0.03 ^b^	0.76 ± 0.01 ^b^
35	2-Ethylfuran	689	296.26	689	1.30	8	7.93 ± 0.22 ^b^	8.36 ± 0.17 ^b^	10.22 ± 0.37 ^a^
36	Butanal	602	224.76	601	1.28	0.0013	3.64 ± 0.06 ^c^	10.20 ± 0.19 ^a^	8.71 ± 0.17 ^b^
37	Propanol	554	193.12	554	1.11	0.24	19.58 ± 0.81 ^c^	26.37 ± 0.50 ^b^	28.33 ± 0.21 ^a^
38	Ethanol	513	169.76	489	1.04	0.62	30.45 ± 1.45 ^a^	31.23 ± 0.22 ^a^	27.05 ± 0.11 ^b^
39	(E)-2-Pentenal M	757	395.21	757	1.10	1.4	2.74 ± 0.34 ^a^	1.15 ± 0.053 ^b^	1.57 ± 0.03 ^b^
40	(E)-2-Pentenal D	765	409.22	757	1.36	1.4	1.62 ± 0.35 ^a^	0.26 ± 0.01 ^b^	0.35 ± 0.01 ^b^
41	Heptanal M	902	645.18	901	1.35	0.26	1.95 ± 0.07 ^a^	1.20 ± 0.02 ^b^	1.10 ± 0.03 ^b^
42	Heptanal D	902	644.16	901	1.68	0.26	0.68 ± 0.04 ^a^	0.29 ± 0.01 ^b^	0.25 ± 0.01 ^b^
43	Nonanal	1075	926.58	1085	1.46	0.0031	10.74 ± 0.03 ^a^	10.4 ± 0.01 ^a^	10.44 ± 0.01 ^a^
44	1-Phenylethanone M	1057	894.48	1058	1.16	0.0012	1.02 ± 0.05 ^a^	0.70 ± 0.03 ^b^	0.66 ± 0.01 ^b^
45	1-Phenylethanone D	1056	891.23	1058	1.56	0.0012	0.18 ± 0.01 ^a^	0.18 ± 0.01 ^b^	0.19 ± 0.01 ^b^
46	α-Ionone M	1350	1583.50	1418	1.53	0.0016	2.19 ± 0.13 ^b^	1.32 ± 0.02 ^b^	1.23 ± 0.04 ^a^
47	α-Ionone D	1348	1579.10	1418	2.20	0.0016	0.57 ± 0.03 ^a^	0.63 ± 0.01 ^b^	0.54 ± 0.05 ^b^
48	2(5H)-Furanone, 3-hydroxy-4,5-dimethyl-M	1098	968.68	1099	1.22	0.0009	1.28 ± 0.01 ^b^	1.41 ± 0.04 ^a^	1.40 ± 0.01 ^b^
49	2(5*H*)-Furanone, 3-hydroxy-4,5-dimethyl D	1100	973.21	1099	1.60	0.0009	0.80 ± 0.01 ^b^	0.89 ± 0.04 ^a^	0.85 ± 0.01 ^a^
50	Phenylacetaldehyde M	1052	884.86	1050	1.26	0.00072	0.28 ± 0.06 ^c^	0.80 ± 0.04 ^a^	0.53 ± 0.01 ^b^
51	Phenylacetaldehyde D	1051	882.63	1050	1.54	0.00072	0.05 ± 0.01 ^c^	0.16 ± 0.01 ^a^	0.09 ± 0.01 ^b^
52	α-Phellandrene M	1007	810.57	1004	1.22	3.5	0.87 ± 0.04 ^a^	0.25 ± 0.01 ^b^	0.23 ± 0.01 ^b^
53	α-Phellandrene D	1004	805.29	1004	1.68	3.5	0.20 ± 0.01 ^a^	0.22 ± 0.01 ^a^	0.20 ± 0.02 ^a^
54	Hexanoic acid	992	785.77	997	1.31	0.0031	0.42 ± 0.11 ^a^	0.22 ± 0.01 ^b^	0.17 ± 0.01 ^b^
55	2-Phenylethanol M	1121	1013.94	1119	1.29	0.0017	1.39 ± 0.59 ^a^	0.16 ± 0.01 ^b^	0.14 ± 0.01 ^b^
56	2-Phenylethanol D	1121	1012.92	1119	1.51	0.0017	0.27 ± 0.12 ^a^	0.10 ± 0.01 ^a^	0.09 ± 0.01 ^a^
57	Benzene acetic acid methyl ester D	1145	1060.53	1176	1.65	50	0.27 ± 0.01 ^a^	0.31 ± 0.01 ^a^	0.31 ± 0.01 ^a^
58	Benzene acetic acid methyl ester M	1147	1065.59	1176	1.26	50	0.54 ± 0.01 ^a^	0.25 ± 0.01 ^a^	0.30 ± 0.01 ^a^

^1.^ RI, Retention indices calculated on a DB-5MS column against n-alkanes; RT, retention time (s); Dt, relative drift time; RI’: theoretical value reported from NIST Chemistry WebBook. ^2.^ na, this information is not available. ^3.^ Different superscript letters in the same row denote significant differences (Duncan, *p* < 0.05). ^4.^ Substance suffixes M and D are monomers and dimers of the same substance, respectively.

**Table 2:** Total volatile compounds of pandan leaves under different processes identified by HS-SPME-GC-O-MS.

No.	Compound ^1^	CAS	Formula	MW	RI ^2^	RT [min]	RI’ ^2^	Relative Concentration (mg/kg) ^3^			FEMA ^4^
								T1-1	T1-2	T1-3	
1	Isovaleraldehyde	590-86-3	C_5_H_10_O	96.13	688	2.05	649	- ^a^	0.012 ± 0.0071 ^b^	- ^a^	2692
2	2-Methylbutyraldehyde	96-17-3	C_5_H_10_O	86.13	691	2.13	659	- ^a^	0.074 ± 0.012 ^b^	- ^a^	2691
3	3-Methyl-3-buten-2-one	814-78-8	C_5_H_8_O	84.12	693	2.19	653	- ^a^	- ^a^	0.14 ± 0.013 ^b^	na
4	1-Penten-3-ol	616-25-1	C_5_H_10_O	86.13	697	2.32	673	0.12 ± 0.031 ^b^	- ^a^	- ^a^	3584
5	2-Ethylfuran	3208-16-0	C_6_H_8_O	96.13	703	2.48	702	0.077 ± 0.021 ^b^	- ^a^	0.049 ± 0.0056 ^b^	3673
6	Trans-2-Pentenal	1576-87-0	C_5_H_8_O	84.12	738	3.49	754	0.043 ± 0.0087 ^c^	- ^a^	0.12 ± 0.021 ^b^	3218
7	Cis-2-Pentenol	1576-95-0	C_5_H_10_O	86.13	750	3.86	769	0.18 ± 0.035 ^b^	- ^a^	- ^a^	4305
8	Hexanal	66-25-1	C_6_H_12_O	100.16	779	4.69	799	0.10 ± 0.0051 ^a^	0.047 ± 0.012 ^b^	0.059 ± 0.011 ^b^	2557
9	Hex-2-enal	505-57-7	C_6_H_10_O	98.14	835	6.34	848	0.19 ± 0.045 ^b^	- ^a^	- ^a^	2560
10	Trans-2-Hexenal	6728-26-3	C_6_H_10_O	98.14	835	6.34	854	0.19 ± 0.045 ^b^	- ^a^	0.036 ± 0.0024 ^c^	2560
11	M-Xylene	108-38-3	C_8_H_10_	106.17	851	6.77	861	- ^a^	0.076 ± 0.019 ^b^	0.045 ± 0.012 ^b^	na
12	1-Hexanol	111-27-3	C_6_H_14_O	102.17	854	6.90	867	0.11 ± 0.0088 ^b^	- ^a^	- ^a^	2567
13	Styrene	100-42-5	C_8_H_8_	104.15	874	7.47	890	0.048 ± 0.012 ^c^	0.13 ± 0.024 ^b^	- ^a^	na
14	Butyl acrylate	141-32-2	C_7_H_12_O_2_	128.17	883	7.73	902	0.031 ± 0.0062 ^b^	- ^a^	- ^a^	na
15	(Z)-4-Heptenal	6728-31-0	C_7_H_12_O	112.17	886	7.81	897	0.069 ± 0.007 ^b^	- ^a^	- ^a^	3289
16	Ethyl valerate	539-82-2	C_7_H_14_O_2_	130.18	888	7.86	898	- ^a^	0.059 ± 0.011 ^b^	- ^a^	2462
17	3-(Methylthio)propionaldehyde	3268-49-3	C_4_H_8_OS	104.17	894	8.05	911	- ^a^	0.064 ± 0.012 ^b^	0.072 ± 0.009 ^b^	2747
18	Benzaldehyde	100-52-7	C_7_H_6_O	106.12	952	9.75	961	0.65 ± 0.056 ^b^	- ^a^	0.55 ± 0.080 ^b^	2127
19	3-Methyl-2(5H)-furanone	22122-36-7	C_5_H_6_O_2_	98.1	967	10.16	982	0.11 ± 0.0072 ^b^	- ^a^	- ^a^	na
20	6-Methyl-5-hepten-2-one	110-93-0	C_8_H_14_O	126.2	979	10.51	988	- ^a^	0.12 ± 0.019 ^b^	- ^a^	2707
21	Myrcene	123-35-3	C_10_H_16_	136.23	983	10.63	992	- ^a^	0.084 ± 0.0054 ^b^	- ^a^	2762
22	2-Octanone	111-13-7	C_8_H_16_O	128.21	985	10.72	992	0.65 ± 0.033 ^b^	0.13 ± 0.017 ^c^	- ^a^	2802
23	Ethyl Hexanoate	123-66-0	C_8_H_16_O_2_	144.21	994	10.95	996	- ^a^	0.13 ± 0.020 ^b^	- ^a^	2439
24	(*+*)-Dipentene	5989-27-5	C_10_H_16_	136.23	1024	11.82	1002	0.18 ± 0.049 ^c^	0.70 ± 0.093 ^b^	- ^a^	2633
25	Benzyl alcohol	100-51-6	C_7_H_8_O	108.14	1032	12.07	1033	- ^a^	0.096 ± 0.013 ^b^	0.060 ± 0.016 ^b^	2137
26	Phenylacetaldehyde	122-78-1	C_8_H_8_O	120.15	1040	12.32	1043	0.080 ± 0.0043 ^c^	0.10 ± 0.0078 ^b^	0.10 ± 0.0052 ^b^	2874
27	Ocimene	13877-91-3	C_10_H_16_	136.23	1041	12.33	1044	- ^a^	0.14 ± 0.012 ^b^	- ^a^	3539
28	Isophorone	78-59-1	C_9_H_14_O	138.21	1055	12.73	1124	- ^a^	- ^a^	0.094 ± 0.0032 ^b^	3553
29	(E)-2-Octenal	2548-87-0	C_8_H_14_O	126.20	1056	12.74	1057	0.24 ± 0.041 ^b^	- ^a^	- ^a^	3215
30	2-Acetyl pyrrole	1072-83-9	C_6_H_7_NO	109.13	1060	12.87	1063	- ^a^	- ^a^	0.11 ± 0.0009 ^b^	3202
31	Acetophenone	98-86-2	C_8_H_8_O	120.15	1061	12.88	1065	0.052 ± 0.007 ^c^	0.27 ± 0.052 ^b^	0.086 ± 0.006 ^c^	2009
32	(3E,5E)-octa-3,5-dien-2-one	38284-27-4	C_8_H_12_O	124.18	1065	13.02	1081	- ^a^	0.088 ± 0.014 ^c^	0.14 ± 0.021 ^b^	na
33	1-Octanol	111-87-5	C_8_H_18_O	130.23	1069	13.13	1068	0.31 ± 0.084 ^b^	- ^a^	- ^a^	2800
34	Terpinolene	586-62-9	C_10_H_16_	136.23	1078	13.39	1083	- ^a^	0.068 ± 0.015 ^b^	- ^a^	3046
35	α-Naginatene	15186-51-3	C_10_H_14_O	150.22	1085	13.60	1093	- ^a^	0.031 ± 0.0064 ^b^	- ^a^	4174
36	Methyl benzoate	93-58-3	C_8_H_8_O_2_	136.15	1088	13.68	1093	- ^a^	0.092 ± 0.023 ^b^	- ^a^	2683
37	Ethyl heptanoate	106-30-9	C_9_H_18_O_2_	158.24	1091	13.79	1095	- ^a^	0.067 ± 0.010 ^b^	- ^a^	2437
38	Nonanal	124-19-6	C_9_H_18_O	142.24	1099	14.01	1104	0.40 ± 0.10 ^b^	0.23 ± 0.049 ^b^	0.41 ± 0.13 ^b^	2782
39	Phenethyl alcohol	60-12-8	C_8_H_10_O	122.16	1115	14.22	1121	0.27 ± 0.03 ^b^	- ^a^	- ^a^	2858
40	1,2,4,5-Tetramethylbenzene	95-93-2	C_10_H_14_	134.22	1130	14.33	1131	0.082 ± 0.014 ^c^	0.15 ± 0.0094 ^b^	- ^a^	na
41	(2E,6E)-nona-2,6-dienal	17587-33-6	C_9_H_14_O	138.21	1145	15.34	1153	0.23 ± 0.027 ^b^	- ^a^	- ^a^	3766
42	Ethyl benzoate	93-89-0	C_9_H_10_O_2_	150.17	1165	15.79	1170	- ^a^	0.043 ± 0.0061 ^b^	- ^a^	2422
43	DL-Menthol	1490-04-6	C_10_H_20_O	156.27	1170	16.07	1173	0.49 ± 0.19 ^b^	- ^a^	- ^a^	2665
44	Naphthalene	91-20-3	C_10_H_8_	128.17	1173	16.18	1178	- ^a^	0.20 ± 0.063 ^b^	- ^a^	na
45	4′-Methylacetophenone	122-00-9	C_9_H_10_O	134.18	1175	16.22	1183	- ^a^	0.050 ± 0.013 ^b^	- ^a^	2677
46	Ethyl (Z)-oct-4-enoate	34495-71-1	C_10_H_18_O_2_	170.25	1178	16.30	1187	- ^a^	0.073 ± 0.0086 ^b^	- ^a^	3344
47	Ethyl caprylate	106-32-1	C_10_H_20_O_2_	172.26	1187	16.57	1193	0.067 ± 0.011 ^c^	0.68 ± 0.15 ^b^	0.83 ± 0.15 ^b^	2449
48	Decyl aldehyde	112-31-2	C_10_H_20_O	156.27	1197	16.87	1200	0.14 ± 0.051 ^b^	0.050 ± 0.015 ^b^	- ^a^	2362
49	β-Cyclocitral	432-25-7	C_10_H_16_O	152.23	1210	17.23	1214	0.24 ± 0.019 ^b^	0.082 ± 0.007 ^c^	0.099 ± 0.014 ^c^	3639
50	2,6,6-Trimethyl-1-Cyclohexene-1-acetaldehyde	472-66-2	C_11_H_18_O	166.26	1246	18.28	1251	0.037 ± 0.0099 ^b^	- ^a^	- ^a^	3474
51	Citral	5392-40-5	C_10_H_16_O	152.23	1258	18.65	1260	- ^a^	0.071 ± 0.0089 ^b^	- ^a^	2303
52	Ethyl 2-hydroxybenzoate	118-61-6	C_9_H_10_O_3_	166.17	1259	18.66	1270	0.074 ± 0.007 ^b^	- ^a^	- ^a^	2458
53	1-Phenylethyl propionate	120-45-6	C_11_H_14_O_2_	178.23	1269	18.95	1275	- ^a^	0.041 ± 0.0068 ^b^	- ^a^	2689
54	Nonanoic acid	112-05-0	C_9_H_18_O_2_	158.24	1272	19.05	1280	0.43 ± 0.024 ^b^	- ^a^	- ^a^	2784
55	Ethyl nonanoate	123-29-5	C_11_H_22_O_2_	186.29	1287	19.50	1294	0.061 ± 0.0076 ^c^	0.20 ± 0.045 ^b^	0.073 ± 0.017 ^c^	2447
56	Gamma-Nonanolactone	104-61-0	C_9_H_16_O_2_	156.22	1353	21.41	1360	0.027 ± 0.0072 ^b^	- ^a^	- ^a^	2781
57	Alpha-Copaene	3856-25-5	C_15_H_24_	204.35	1369	21.88	1376	0.052 ± 0.0069 ^b^	- ^a^	- ^a^	2902
58	Ethyl caprate	110-38-3	C_12_H_24_O_2_	200.32	1391	22.50	1397	0.10 ± 0.012 ^b^	0.85 ± 0.29 ^b^	0.68 ± 0.25 ^b^	2432
59	α-Ionone	127-41-3	C_13_H_20_O	192.30	1418	23.30	1427	0.50 ± 0.028 ^a^	0.090 ± 0.0093 ^c^	0.097 ± 0.0055 ^c^	2594
60	6,10-Dimethyl-5,9-undecadien-2-one	689-67-8	C_13_H_22_O	194.31	1444	24.07	1451	0.16 ± 0.032 ^b^	0.15 ± 0.0028 ^b^	0.076 ± 0.0099 ^c^	3542
61	3-Buten-2-one	14901-07-6	C_13_H_20_O	192.30	1475	24.97	1488	- ^a^	0.11 ± 0.005 ^c^	- ^a^	2594
62	Ethyl Undecanoate	627-90-7	C_13_H_26_O_2_	214.34	1492	25.46	1496	- ^a^	0.30 ± 0.12 ^b^	0.033 ± 0.0056 ^b^	3492
63	Butylated Hydroxytoluene	128-37-0	C_15_H_24_O	220.35	1500	25.67	1509	- ^a^	0.27 ± 0.0087 ^b^	- ^a^	2184
64	β-bisabolene	495-61-4	C_15_H_24_	204.35	1505	25.84	1511	- ^a^	- ^a^	0.033 ± 0.011 ^b^	na
65	Delta-Cadinene	483-76-1	C_15_H_24_	204.35	1515	26.12	1519	0.070 ± 0.0098 ^b^	- ^a^	- ^a^	na
66	Dihydroactinidiolide	17092-92-1	C_11_H_16_O_2_	180.24	1525	26.40	1525	0.28 ± 0.097 ^a^	0.058 ± 0.0067 ^b^	0.042 ± 0.002 ^b^	4020
67	Ethyl laurate	106-33-2	C_14_H_28_O_2_	228.37	1593	28.40	1597	0.41 ± 0.12 ^b^	0.41 ± 0.16 ^b^	0.21 ± 0.061 ^b^	2441
68	Pentadecanal	2765-11-9	C_15_H_30_O	226.40	1712	30.95	1716	0.039 ± 0.0081 ^b^	- ^a^	- ^a^	na
69	Ethyl myristate	124-06-1	C_16_H_32_O_2_	256.42	1782	33.91	1793	0.019 ± 0.0035 ^c^	0.10 ± 0.020 ^b^	0.046 ± 0.0052 ^c^	2445
70	Ethyl pentadecanoate	41114-00-5	C_17_H_34_O_2_	270.45	1871	36.48	1874	0.026 ± 0.0046 ^c^	- ^a^	0.053 ± 0.0051 ^b^	na
71	Palmitic acid ethyl ester	628-97-7	C_18_H_36_O_2_	284.48	1966	38.96	1978	0.16 ± 0.017 ^b^	0.18 ± 0.063 ^ab^	0.2513 ± 0.029 ^a^	2451
72	Ethyl Linoleate	544-35-4	C_20_H_36_O_2_	308.50	2088	42.79	2144	0.028 ± 0.0073 ^b^	- ^a^	0.054 ± 0.013 ^b^	na

^1.^ Compound description reported from Scifinder (American Chemical Society), and the description of the FEMA flavor library. ^2.^ RI: calculated value; RI’: theoretical value reported from the NIST Chemistry WebBook, https://doi.org/10.18434/T4D303 (accessed on 11 February 2025). ^3.^ Different superscript letters in the same row denote significant differences (Ducan, *p* < 0.05). ^4.^ na: these compound data are not available.

**Table 3:** Odor-active compounds identified in pandan leaves by HS-SPME-GC-O-MS and HS-GC-IMS (based on OAV > 1).

No.	Compounds	Identification ^1^	FD ^2^			OAV			Odor Series ^3^
			T1-1	T1-2	T1-3	T1-1	T1-2	T1-3	
1	Isovaleraldehyde	MS, RI, O, S	-	256	-	- ^a^	123 ± 71 ^b^	- ^a^	6, 12
2	2-Methylbutyraldehyde	MS, RI, O, S	-	32	-	- ^a^	3.73 ± 0.61 ^b^	- ^a^	1, 13
3	Hexanal	MS, IMS, RI, O, S	256	128	256	73.71 ± 3.64 ^a^	33.86 ± 8.29 ^b^	42.07 ± 8.21 ^b^	2, 4, 6
4	Hex-2-enal	MS, IMS, RI, O, S	8	-	-	6.28 ± 1.49 ^b^	- ^a^	- ^a^	1, 8
5	Trans-2-Hexenal	MS, RI, O, S	64	-	8	6.28 ± 1.49 ^b^	- ^a^	1.22 ± 0.08 ^c^	6, 8
6	(Z)-4-Heptenal	MS, RI, O, S	64	-	-	20.32 ± 2.06 ^b^	- ^a^	- ^a^	2, 8
7	3-(Methylthio)propionaldehyde	MS, RI, O, S	-	512	512	- ^a^	1012.7 ± 192.06 ^b^	1136.51 ± 142.86 ^b^	7, 11
8	Benzaldehyde	MS, IMS, RI, O, S	8	-	8	6.49 ± 0.56 ^b^	- ^a^	5.52 ± 0.8 ^b^	1,6, 7, 12, 14
9	Phenylacetaldehyde	MS, RI, O, S	128	128	256	66.25 ± 3.58 ^c^	86.92 ± 6.5 ^b^	85.25 ± 4.33 ^b^	5, 6, 10, 12
10	(E)-2-Octenal	MS, RI, O, S	16	-	-	19.67 ± 3.39 ^b^	- ^a^	- ^a^	2, 4, 6, 8, 10
11	1-Nonanal	MS, IMS, RI, O, S	256	256	512	198.1 ± 50.6 ^b^	114.85 ± 24.5 ^b^	203.25 ± 67.55 ^b^	2, 5, 6, 8
12	(2E,6E)-nona-2,6-dienal	MS, RI, O, S	128	-	-	167.71 ± 19.57 ^b^	- ^a^	- ^a^	6, 8
13	Decyl aldehyde	MS, RI, O, S	64	8	-	52.12 ± 19.73 ^b^	19.35 ± 5.81 ^b^	- ^a^	2, 5, 6
14	β-Cyclocitral	MS, RI, O, S	32	16	8	12.63 ± 0.96 ^b^	4.27 ± 0.36 ^c^	5.14 ± 0.72 ^c^	5, 9, 12
15	Citral	MS, RI, O, S	-	8	-	- ^a^	1.19 ± 0.15 ^b^	- ^a^	6
16	Butanal	IMS, RI, O, S	1	4	4	2.80 ± 0.04 ^c^	7.84 ± 0.15 ^a^	6.70 ± 0.13 ^b^	6, 8, 10
17	Alcohols 1-Penten-3-ol	MS, IMS, RI, O, S	16	-	-	12.38 ± 3.12 ^b^	- ^a^	- ^a^	2, 4
18	1-Hexanol	MS, IMS, RI, O, S	32	-	-	3.26 ± 0.26 ^b^	- ^a^	- ^a^	5, 9, 12
19	1-Octanol	MS, RI, O, S	8	-	-	14.28 ± 3.82 ^b^	- ^a^	- ^a^	2, 3, 5, 7, 15
20	Phenethyl alcohol	MS, IMS, RI, O, S	64	-	-	22.34 ± 2.50 ^b^	- ^a^	- ^a^	5, 6, 12
					-				
21	Esters Butyl acrylate	MS, RI, O, S	128	-	-	56.55 ± 11.27 ^b^	- ^a^	- ^a^	6
22	Ethyl valerate	MS, RI, O, S	-	256	-	- ^a^	101.72 ± 19.83 ^b^	- ^a^	6, 9
23	Ethyl Hexanoate	MS, RI, O, S	-	64	-	- ^a^	42.13 ± 6.77 ^b^	- ^a^	6, 12
24	Methyl benzoate	MS, RI, O, S	-	128	-	- ^a^	61.53 ± 15.53 ^b^	- ^a^	5, 6, 8, 9
25	Ethyl caprylate	MS, RI, O, S	4	8	64	3.06 ± 0.48 ^c^	30.72 ± 6.67 ^b^	37.83 ± 6.74 ^b^	5, 6
26	Ethyl 2-hydroxybenzoate	MS, RI, O, S	32	-	-	14.84 ± 1.4 ^b^	- ^a^	- ^a^	8, 12
27	Ethyl nonanoate	MS, RI, O, S	32	64	8	6.78 ± 0.84 ^c^	21.92 ± 4.99 ^b^	8.11 ± 1.84 ^c^	5, 6
28	Gamma-Nonanolactone	MS, RI, O, S	4	-	-	5.91 ± 1.6 ^b^	- ^a^	- ^a^	6, 12
29	Ethyl caprate	MS, RI, O, S	128	256	256	87.25 ± 10.25 ^b^	712 ± 245.75 ^b^	565.25 ± 205.83 ^b^	1, 6
30	Ethyl Undecanoate	MS, RI, O, S	-	512	128	- ^a^	988.33 ± 397.33 ^b^	111.67 ± 18.67 ^b^	1, 2, 6, 16
31	Ethyl laurate	MS, RI, O, S	128	128	64	205.15 ± 60.7 ^b^	205.55 ± 78.35 ^b^	106.85 ± 30.45 ^b^	5, 6, 8, 12, 15
32	Ketones 2-Octanone	MS, RI, O, S	8	4	-	2.81 ± 0.14 ^b^	0.58 ± 0.08 ^c^	- ^a^	2, 5, 11
33	Isophorone	MS, RI, O, S	-	-	32	- ^a^	- ^a^	55.18 ± 1.88 ^b^	8, 9
34	Acetophenone	MS, RI, O, S	8	32	8	5.18 ± 0.7 ^c^	27.44 ± 5.2 ^b^	8.64 ± 0.6 ^c^	1, 5
35	4′-Methylacetophenone	MS, RI, O, S	-	64	-	- ^a^	24.85 ± 6.55 ^b^	- ^a^	1, 5, 12
36	α-Ionone	MS, IMS, RI, O, S	64	32	32	124.53 ± 6.98 ^a^	22.6 ± 2.33 ^c^	24.2 ± 1.38 ^c^	3, 5
37	3-Buten-2-one	MS, RI, O, S	-	512	-	- ^a^	962.5 ± 41.67 ^c^	- ^a^	3, 5
38	3-Hydroxy-2-Butanone	IMS, RI, O, S	4	1	1	2.43 ± 0.08 ^a^	0.44 ± 0.01 ^b^	0.42 ± 0.01 ^b^	2, 8
39	Alkenes (*+*)-Dipentene	MS, RI, O, S	16	32	-	4.11 ± 1.09 ^c^	15.61 ± 2.08 ^b^	- ^a^	6, 8
40	Terpinolene	MS, RI, O, S		4	-	- ^a^	1.65 ± 0.37 ^b^	- ^a^	3, 6
41	Aromatic compounds 1,2,4,5-Tetramethylbenzene	MS, RI, O, S	4	4	-	0.94 ± 0.16 ^c^	1.72 ± 0.11 ^b^	- ^a^	12, 16
42	Acids Nonanoic acid	MS, RI, O, S	512	-	-	269.31 ± 14.75 ^b^	- ^a^	- ^a^	2, 8, 16
43	Nitrogenous compounds 2-Acetyl-1-pyrroline	IMS, RI, O, S	256	512	512	718.01 ± 27.28 ^b^	1065.24 ± 17.93 ^a^	1080.06 ± 13.22 ^a^	7, 12

^1.^ MS, IMS, RI, O, and S were identified via mass spectrometry, the retention index, olfactometry, and the standard, respectively. ^2.^ The “-” mark indicates that the volatile compound was not sniffed. ^3.^ The odorant series represents the classification of each compound according to its odor description. 1, nutty; 2, oily; 3, woody; 4, grassy; 5, flowery; 6, fruity; 7, roasted; 8, green/vegetative; 9, herby; 10, spicy; 11, mushroomy; 12, sweet; 13, fermented; 14, bitter; 15, balsamic; 16, sour. Different superscript letters in the same row denote significant differences (Duncan, *p* < 0.05).

## Data Availability

The data presented in this study are available on request from the corresponding authors. The data are not publicly available due to privacy restrictions.

## Supplementary Materials

The following supporting information can be downloaded at: https://www.mdpi.com/article/10.3390/foods14060935/s1, References [39,40,41,42,43,44,45,46,47,48,49,50,51,52,53,54,55] are cited in the Supplementary Materials. Figure S1. Shows a three-dimensional spectrum of GC-IMS, where the three axes represent the ion migration time (X-axis), retention time (Y-axis), and signal intensity (Z-axis); Figure S2. Overlapping chromatograms; Figure S3. Qualitative GC-IMS spectra of volatile components in pandan leaves; Figure S4. Loading Scatter Plot (origin graph in SIMCA); Table S1. The Odor description and compounds of GC-IMS identified volatile compounds from fresh and treated pandan leaves; Table S2. Total volatile compound components in different processes of pandan leaves by HS-SPME-GC-O-MS and AEDA; Table S3. The characterization of 3-(Methylthio)propionaldehyde (Methional); Table S4. Structre of Aroma-active compounds identified by SPME-GC-O-MS and GC-IMS (based on OAV > 1).
