# Supplementary material for: Comprehensive Characterization of Aroma Profile of “Glutinous Rice” Flavor in Pandanus amaryllifolius Roxb. Using HS–SPME–GC–O–MS and HS-GC-IMS Technology Coupled with OAV
Source: Foods. 2025 Mar 10;14(6):935. doi: 10.3390/foods14060935 (PMC11941618; doi:10.3390/foods14060935)
Supplement: Supplementary file 1 [file foods-14-00935-s001.zip › foods-3498157-supplementary.pdf]

foods MDPI

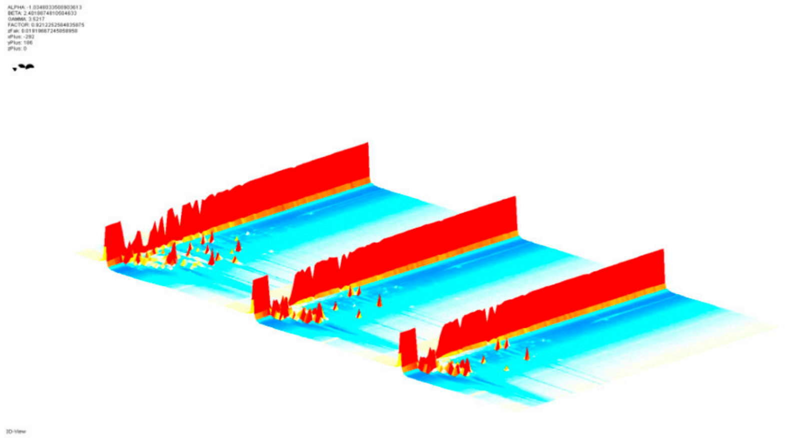

Figure S1. Three-dimensional GC-IMS spectra of volatiles in pandan leaves.

Figure. S1 shows a three-dimensional spectrum of GC-IMS, where the three axes represent the ion migration time (X-axis), retention time (Y-axis), and signal intensity (Z-axis).

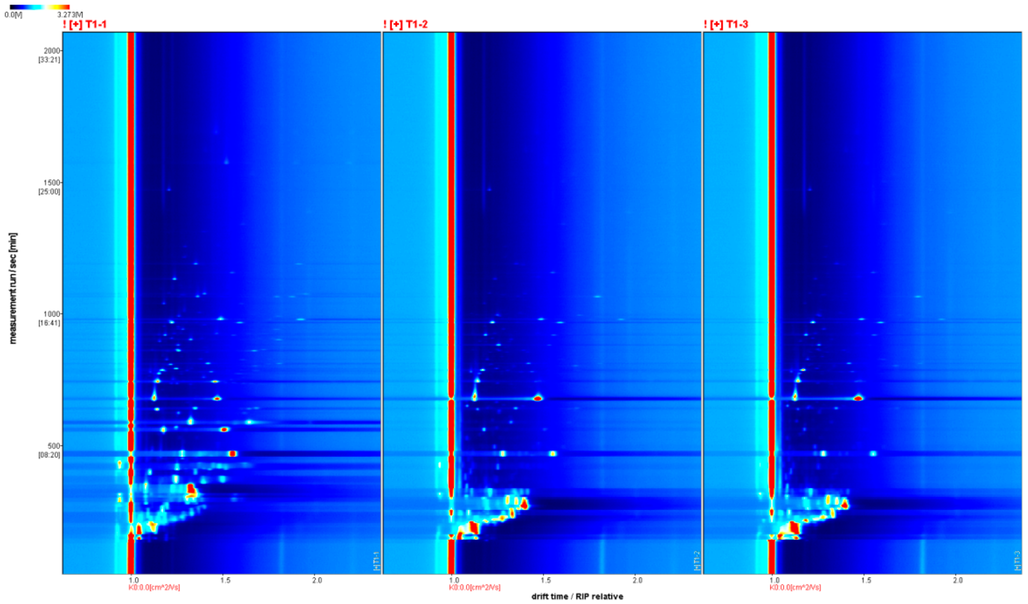

Figure S2. overlapping chromatograms.

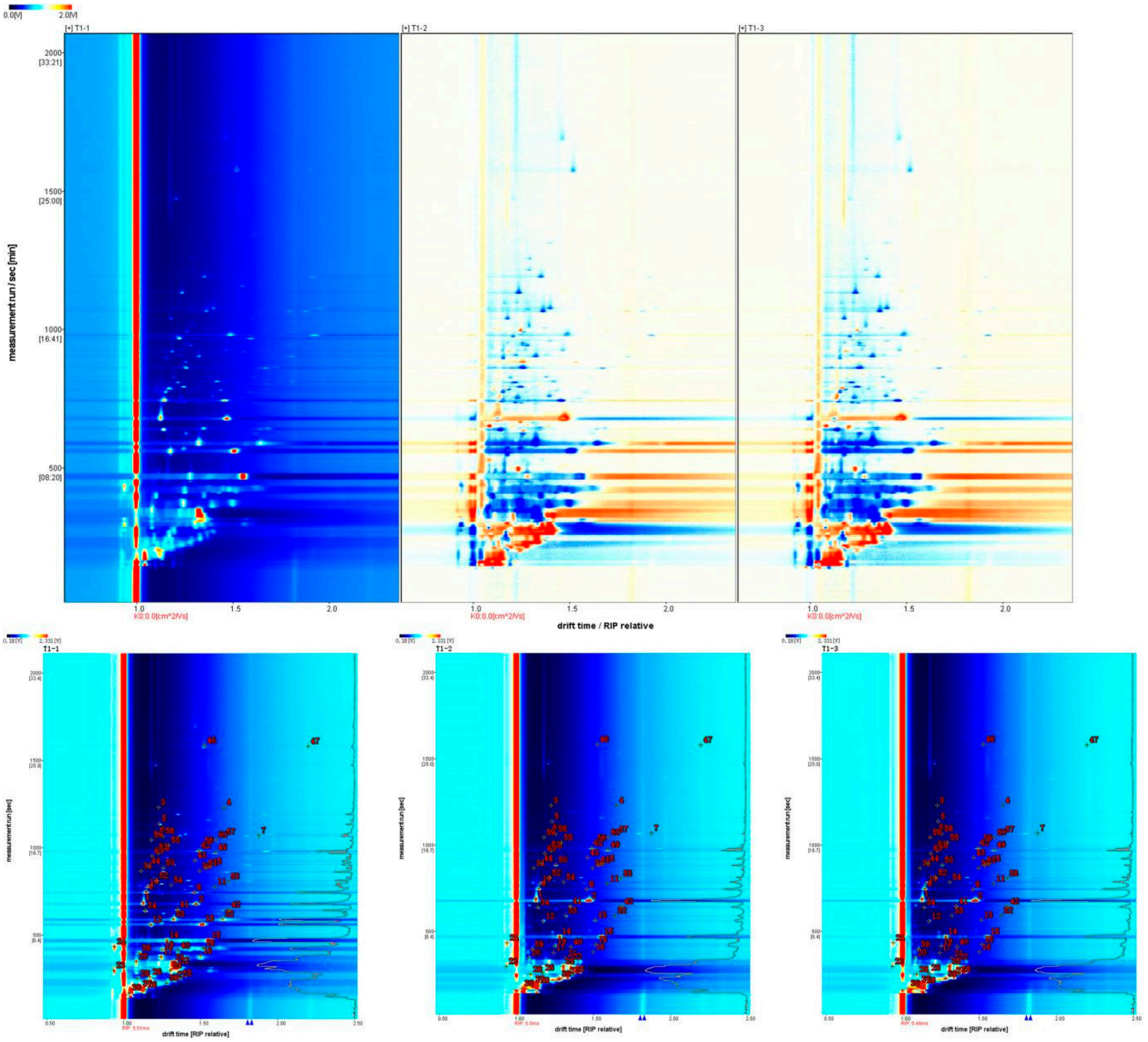

Figure S3. Qualitative GC-IMS spectra of volatile components in pandan leaves.

1.1 The entire background of the figure (Figure. S2) is blue, with a red vertical line at the X-coordinate of 1.0 representing the RIP peak (reactive ion peak, normalized). The Y-axis indicates the retention time of the gas-phase substances (in seconds), while the X-axis represents the relative migration time (normalized, a.u.). Each point adjacent to the RIP peak represents a specific volatile organic compound (VOC). The color intensity, ranging from blue to red, indicates the peak intensity, with darker shades signifying higher peak intensities.

To further facilitate a visual comparison of the differences in volatile components, the spectrum of sample T1-1 was chosen as a reference. The spectra of the other samples then subtracted from reference to obtain differential comparison maps, as shown in Figure. S3. If the content of volatile organic compounds in the target sample is the same as in the reference the resulting background will appear white. In contrast, red indicates that the concentration of a particular substance is higher in the target sample compared to the reference, while blue signifies that the concentration of that substance is lower in the target sample than in the reference.And the numbers represent the peak signals of the corresponding substances detected.

1.2 During GC-IMS analyses, volatile compounds were evaporated from the heated sample and then transferredinto the ionization zone of the IMS drift tube via carrier gas, in which various ions were generated with different drift times [39,15]. in the ionization region, certain compounds may produce multiple signals, forming monomers, dimersor

even trimers. and this was mainly dependent on the concentrations of volatile substances and their halflife in thedrift tube [39,40]. The presence of dimers may correlate with specific sensory attributes. However, in the current manuscript, multiple signals from the same compound were detected. Notably, flavor annotation in this study was performed at the compound level rather than the signal level. As the Ion Mobility Spectrometry (IMS) system lacks a sniffing port, to address this limitation, flavor interpretation was conducted using the FEMA Flavor Library and Flavor-Base 10, as detailed in the footnote of Table 1.

Ions are formed in mobility spectrometers mostly through chemical reactions between sample molecules and a reservoir of ions known as reactant ions. These reactions are affected by the properties of particular molecules; thus, the very first step in response adds a layer of selectivity in addition to the mobility characterization. Reactant ions, which are formed through beta emitters in air at ambient pressure and in the absence of a regent gas, are in positive polarity H+(H2O)n and in negative polarity O2-(H2O)n。Sample or analyte molecules M are ionized in positive polarity through collisions with hydrated protons, forming a cluster ion (Equation 1.4), which is stabilized through the displacement of adducted water, yielding a production, a protonated monomer:

Introduction to Ion Mobility Spectrometry

$$M + H^+(H_2O)_n \leftrightarrow MH^+(H_2O)_n^* \leftrightarrow MH^+(H_2O)_{n-x} + xH_2O \qquad (1.4)$$

Sample neutral + Reactant ion　　Cluster ion　　Product ion

Protonated monomer + Water

Should the vapor concentration of M in the reaction region increase further, a second product ion can be formed as another sample neutral attaches to the protonated monomer, displacing a water molecule and yielding a proton-bound dimer M2H+-(H2O)n-x:

$$MH^+(H_2O)_n + M \leftrightarrow M_2H^+(H_2O)_{n-x} + xH_2O \qquad (1.5)$$

Protonated monomer + Sample　　Proton bound dimer + water

While formation of proton-bound trimers and tetramers may occur in the vaporrich region of the ion source, these ions have short lifetimes when extracted into the purified atmosphere of a drift region and are rarely observed in mobility spectra at ambient temperatures or above.

Table S1. The Odor description and compounds of GC-IMS identified volatile compounds from fresh and treated pandan leaves.

No.	Compound [1]	CAS Numbers	Formula	Mol. Weight	FEMA [2]	Odor [3]
1	2-acetyl-1-pyrroline	85213-22-5	C6H9NO	111.1	4249	Popcorn、Savory
2	Geraniol	106-24-1	C10H18O	154.3	2507	Floral，Fruit
3	Estragole	140-67-0	C10H12O	148.2	2411	Anisic
4	Borneol	507-70-0	C10H18O	154.3	2157	Balsamic、Camphor, Fragrant, Green

No.	Compound [1]	CAS Numbers	Formula	Mol. Weight	FEMA [2]	Odor [3]
5	Benzaldehyde	100-52-7	C_7H_6O	106.1	2127	Bitter Almond, Burnt Sugar, Cherry, Malt
6	1-Octen-3-ol	3391-86-4	$C_8H_{16}O$	128.2	2805	Cucumber, Earthy, Fat, Floral, Mushroom
7	2-Hexenal	505-57-7	$C_6H_{10}O$	98.1	2560	Fruit, Green
8	Hexanal	66-25-1	$C_6H_{12}O$	100.2	2557	Apple, Fat, Green, Oil
9	3-Methylbutanol	123-51-3	$C_5H_{12}O$	88.1	2057	Fermented, Burnt, Cocoa, Floral, Malt
10	Pentanol	71-41-0	$C_5H_{12}O$	88.1	2056	Fermented, Balsamic, Fruit, Green, Pungent, Yeast
11	3-Hydroxy-2-Butanone	513-86-0	$C_4H_8O_2$	88.1	2008	Butter, Creamy, Green Pepper
12	1-Penten-3-ol	616-25-1	$C_5H_{10}O$	86.1	3584	Buttery, Grassy
13	(*Z*)-2-Pentenol	1576-95-0	$C_5H_{10}O$	86.1	4305	Fruity、Green
14	2-Methylpropanol	78-83-1	$C_4H_{10}O$	74.1	2179	Ethereal、Wine
15	Ethyl Acetate	141-78-6	$C_4H_8O_2$	88.1	2414	Fruity、Sweet
16	2-Methyl-2-propanol	75-65-0	$C_4H_{10}O$	74.1	*na*	Pungent、Camphor
17	2-Acetylpyrrole	1072-83-9	C_6H_7NO	109.1	3202	Bread, Cocoa, Hazelnut, Licorice, Walnut
18	Hexanol	111-27-3	$C_6H_{14}O$	102.2	2567	Herbal、Floral, Sweet
19	2-Methyl-3-furanthiol	28588-74-1	C_5H_6OS	114.2	3188	Fried, Nut, Potato, Roasted Meat
20	2-Ethylfuran	3208-16-0	C_6H_8O	96.1	3673	Butter, Caramel
21	Butanal	123-72-8	C_4H_8O	72.1	2219	Fruity、Green、Pungent
22	Propanol	71-23-8	C_3H_8O	60.1	2928	Alcohol, Candy, Pungent
23	Ethanol	64-17-5	C_2H_6O	46.1	2419	Alcohol, Pungent
24	(*E*)-2-Pentenal	1576-87-0	C_5H_8O	84.1	3218	Pungent, Green
25	Heptanal	111-71-7	$C_7H_{14}O$	114.2	2540	Citrus, Fat, Green, Nut

No.	Compound [1]	CAS Numbers	Formula	Mol. Weight	FEMA [2]	Odor [3]
26	Nonanal	124-19-6	C9H18O	142.2	2782	Fat, Floral, Green, Lemon
27	1-Phenylethanone	98-86-2	C9H18O	142.2	2009	Almonds, Flower, Meat, Must
28	α-Ionone	127-41-3	C13H20O	192.3	2594	Violet, Wood
29	2(5H)-Furanone, 3-hydroxy-4,5-	28664-35-9	C6H8O3	128.1	3634	Caramellike, Maple, Savory, Spice
30	Phenylacetaldehyde	122-78-1	C8H8O	120.2	2874	Berry, Geranium, Honey, Pungent
31	α-Phellandrene	99-83-2	C10H16	136.2	2856	Citrus, Fresh, Pepper, Spice
32	Hexanoic acid	142-62-1	C6H12O2	116.2	2559	Cheese, Oil, Pungent, Sour
33	2-Phenylethanol	60-12-8	C8H10O	122.2	2858	Fruit, Honey, Lilac, Rose
34	Benzene acetic acid methyl ester	101-41-7	C9H10O2	150.2	2733	Honey, Jasmine
35	1	unidentified	-	-	na	-
36	2	unidentified	-	-	na	-
37	3	unidentified	-	-	na	-
38	4	unidentified	-	-	na	-
39	5	unidentified	-	-	na	-

1 Five substances detected by GC-IMS cannot be identifed due to database limitations. 2 na this information is not available. 3 Compounds description reported from Scifinder (American Chemical Society), Odor description of FEMA flavor library, and Flavor-Base 10.

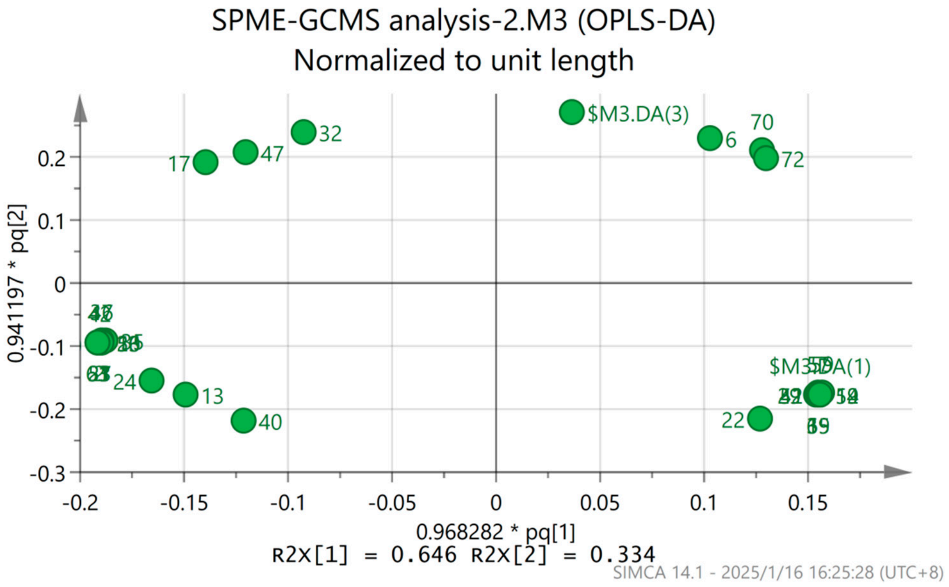

Figure S4. Loading Scatter Plot (origin graph in SIMCA).

1.3. Key AEDA-identified aroma-active compounds

Volatiles from the SPME extracts were analyzed using the AEDA sniffing dilution test to determine the volatile-compound contributions to the overall aromas of the pandan leaves subjected to different processes. As shown in Table S2, an AEDA sniffing test for 72 compounds was performed based on the stationary-phase RIs, compound-standard RTs, and gradient dilutions utilizing the above GC-O-MS conditions. Diluted T1-1, T1-2, and T1-3 extracts were used and a total of 58 aroma-active compounds were identified. Among these, 38 aroma components were identified in the T1-1 sample. Nonanoic acid (512, cheese), a low-carbon-chain fatty acid usually found in the amino acid metabolic pathway, presented the highest flavor sparsity.

In addition, aldehydes such as nonanal (256, green, bean), hexanal (256, fresh grass, fruit), (*2E,6E*)-nona-2,6-dienal (128, green, fruit), and phenylacetaldehyde (128, geranium, honey) were the most abundant and exhibited relatively high FD values, followed by ketones and esters.

For T1-2, only 35 aroma components were identified, among which the aroma compounds with the highest FDs were 3-(methylthio) propionaldehyde (512, cooked potato, mushroomy) and 3-buten-2-one (512, floral, woody, violet). Interestingly, although only 21 aroma components were identified in T1-3, 3-(methylthio) propionaldehyde (512, cooked potato, mushroomy) was again the aroma substance with the highest FD. Most sulfur-containing compounds with low detection thresholds and strong organoleptic properties are integral components of many food flavor profiles [41–43]. 3-methylthiopropionaldehyde, a volatile flavor substance, has been identified for the first time in this experiment. Characterization and description of its taste revealed a strong roasted and fresh mushroom-like aroma. It is speculated that this substance was produced during leaf processing and acts as a basetone of aroma. In this study, in addition to this interesting finding, which were also discovered that ketones and esters dominate the remaining substances, with their FD values slightly lower than 512. Esters are the main volatile compounds found in most fresh plants and fruits and are often associated with fruity and sweet odors [44], most volatile esters are produced by alcoyltransferase-catalyzed esterification reactions [45]. It has been found that substances expressing active odor in the fresh samples were also expressed in the dry-processed samples. However, there were also many substances with high aroma contributions that were not present in the fresh samples (mostly aliphatic carboxylic acid esters), and these compounds contributed to fruity, sweet, and floral aromas. Examples include ethyl decanoate (256, fruit, coconut), ethyl laurate (64-128, floral, sweet, waxy, fruit), ethyl caprylate (64, pineapple, floral, fruit), and

ethyl undecanoate (32-256, soap, coconut, cognac). Some isoprenoids such as α-violetone, β-violetone, and β-cyclocitral were also identified in this experiment, agreeing with the reported result of a SPME-GC-MS study. These three isoprenoid compounds have floral aromas. For example, 3-buten-2-one is often described as sweet and violet.

In addition to aromatic active ketones and ester compounds, other volatile substances such as isovaleraldehyde, 2-methylbutanal, phenylethanol, decylaldehyde, and benzaldehyde have high FD values and contribute to the overall flavor characteristics. Most branched-chain aldehydes are produced through enzymatic pathways triggered by transaminases and (non-enzymatic) Strecker degradation, such as 3-methylbutanal, 2-methylbutanal, and 2-methylpropanal, which are potent flavor compounds [46]. In many foods, these aldehydes are key flavor compounds. In terms of sensory perception, they are often considered malt-flavored and chocolate-like. The results of this study also revealed many N-aldehydes, which can be obtained through the thermal degradation of fatty acids, just as hexanal is produced through the degradation of oleic acid and linoleic acid, these aldehydes had a unique green and fruity aroma. Phenylethanol provides a pleasant rose sweetness and is an important compound in plant phenylpropanoid metabolism pathways. Acting as an intermediate, phenylethanol plays a crucial role in the formation of various volatile aromatic compounds.

In 2010, Wakte and co-workers utilized SPME to screen for 31 volatile compounds using a GC-FID, and they found 2-AP, 2-hexenal, and nonanal as the major compounds in pandan leaves. Furthermore, the major aromatic constituents are 2-AP (8.52%, percentage of total), hexenal (6.63%), 2-hexenal (21.87%), nonanal (10.50%), 2,6-nonadienal (3.11%), 3-methyl-2(5H)-furanone (3.12%), and phytol (5.28%). The results of the present study also indicate that these substances were present at high levels. To identify the key aroma-causing components of pandan more precisely, GC-MS-O testing were performed to sniff the suspected 2-AP aromatics. This work simultaneously compared standard and diluted samples on a single HP-5MS column and conducted SPME-GC-O-MS analyses to characterize the sample, FD, and RI. For all the samples considered in this experiment, the total 2-AP content was quantified using the internal standard as 0.02–0.03 μg/g, the FD was 256–512, and the RI was 910–922. Pandan-leaf pre-treatment can affect 2-AP content, with the concentration ranging from 40 to 450 μg/kg, and that drying and crushing may disrupt the papillae structure of the epidermal cells on the pandan-leaf surfaces [47]. Because 2-AP is contained in the papillae, a small amount may have been lost during the drying and grinding processes performed in this experiment. Note that stable identification of 2-AP using the single SPME-GC-MS method and following equilibrium extraction with the extraction was difficult.

This work also considered other volatile aromatic compounds identified via olfaction. In fact, many unreported aromatic substances were detected in the pandan leaves using the SPME conditions in combination with GC-MS-O. Esters, aldehydes, and ketones were dominant. Note that amino acids can be converted into esters, aldehydes, acids, and other flavor compounds through dehydrogenases. Deaminases, decarboxylases, and ester synthases are involved in these reactions [48]. The synthesis process is as follows: amino acids generate branched ketone groups through transamination and then generate a series of corresponding aldehydes, esters, and alcohols under the action of decarboxylase, coenzyme A, and alcohol dehydrogenase, thereby producing flavor substances [49]. The abundant amino acids in pandan leaves support these reactions, for example, the published literature has shown that the common free amino acids in pandan leaves are glutamic acid (0.41 mg/g), aspartic acid, threonine, serine, histidine, alanine, and proline (0.12 mg/g) [50]. Furthermore, Wong and co-workers reported that a product formed by heating a mixture of proline or serine glucose at an initial pH of 5.2 for 24 h exhibited a pandan-like aroma [51]. Using glucose as a carbon source and glutamic acid and amino acids such as proline and ornithine as nitrogen sources, 2-AP can be metabolized by microorganisms such as Bacillus cereus [52–54]. Free amino acids, such as proline, glutamic acid, serine, and ornithine, may significantly impact the amino-acid metabolism pathways of aromatic substances such as 2-AP during pandan-leaf cultivation.

Under different processing methods, these compounds may contribute differently to the characteristic aromas of pandan leaf compared to that of a fresh sample. Because of the lack of literature describing the various volatile aroma compounds in differently processed pandan leaves, in this study, this work compared fresh and dried samples for the first time. The FD olfactory results indicate the contributions of the key aroma-active compounds. Therefore, this work could describe the odor-active compounds in the samples and the aroma levels and provide inter-aromatic descriptions. However, problems exist regarding measurement of the aroma-active compound contributions to the system when the highest dilution factor and content are taken as variables. First, the aroma descriptions and aroma-active compound ratings provided by sniffers are subject to human error, which arise from differences in knowledge sensitivity and physical condition [55]. With the GC-O-MS method, aroma-active compounds are determined based on the air matrix, which differs from the dichloromethane solvent matrix used in the sniff test. Therefore, this work combined the substance content with the odor perception threshold, defined as the OAV, to statistically analyze the validation-test results.

Table S2. Total volatile compound components in different processes of pandan leaves by HS-SPME-GC-O-MS and AEDA.

No.	Label [1]	VIP-value	P-Value	Perception threshold (mg/kg) [2]	Flavor dilution（FD) [3]			Odor [4]
					T1-1	T1-2	T1-3	
1	Isovaleraldehyde	0.85	0.040	0.0001	–	256	–	Ethereal, Warm sweet, Fruit
2	2-Methylbutyraldehyde	1.02	0.001	0.02	–	32	–	Almond, Cocoa, Fermented, Hazelnut, Malt
3	3-Methyl-3-buten-2-one	1.14	1.00	1	–	–	–	Pungent, Sweet
4	1-Penten-3-ol	0.97	0.002	0.01	16	–	–	Ethereal, Butter, Grass
5	2-Ethylfuran	0.92	0.003	8	–	–	–	Butter, Nut, Caramel
6	Trans-2-Pentenal	1.10	0.001	1.4	–	–	–	Pungent, Green
7	Cis-2-Pentenol	0.98	0.001	0.72	2	–	–	Fresh, Fruit
8	Hexanal	0.93	0.002	0.0014	256	128	256	Apple, Fat, Fresh, Grass
9	Hex-2-enal	0.99	0.002	0.03	8	–	–	Almond, Green
10	Trans-2-Hexenal	0.96	0.002	0.03	64	–	8	Fruit, Green
11	M-Xylene	0.93	0.003	0.18	–	–	–	Benzene, Aromatic
12	1-Hexanol	1.01	0.001	0.034	32	–	–	Herbal, Floral, Sweet
13	Styrene	1.05	0.006	0.15	16	16	–	Balsamic
14	Butyl acrylate	0.99	0.001	0.00055	128	–	–	Fruit
15	(*Z*)-4-Heptenal	1.01	0.001	0.0034	64	–	–	Green, Creamy
16	Ethyl valerate	1.01	0.001	0.00058	–	256	–	Fruit, Herbal
17	3-(Methylthio)propionaldehyde	1.00	0.001	0.000063	–	512	512	Cooked potato, Soy
18	Benzaldehyde	0.99	0.001	0.1	8	–	8	Bitter Almond, Burnt Sugar, Cherry, Malt, Roasted Pepper
19	3-Methyl-2(5H)-furanone	1.01	0.001	na [3]	8	–	–	Caramel
20	6-Methyl-5-hepten-2-one	1.02	0.001	0.3	–	2	–	Citrus, Fruit
21	Myrcene	1.02	0.001	0.1	–	2	–	Balsamic, Fruit, Geranium, Herbal, Musty
22	2-Octanone	1.04	0.001	0.23	8	4	–	Fat, Fragrant, Mold
23	Ethyl Hexanoate	1.02	0.001	0.003	–	64	–	Fruit, Sweet
24	(+)-Dipentene	1.06	0.001	0.045	16	32	–	Citrus, Mint
25	Benzyl alcohol	0.95	0.001	0.62	–	2	2	Floral, Moss, Green
26	Phenylacetaldehyde	0.92	0.009	0.0012	128	128	256	Berry, Geranium, Honey, Pungent
27	Ocimene	1.02	0.001	na	–	8	–	Citrus, Floral, Herbal
28	Isophorone	1.14	1.00	0.0017	–	–	32	Camphorous

No.	Label [1]	VIP-value	P-Value	Perception threshold (mg/kg) [2]	Flavor dilution（FD）[3]			Odor [4]
					T1-1	T1-2	T1-3	
29	(*E*)-2-Octenal	1.00	0.001	0.012	16	–	–	Dandelion, Fat, Fruit, Grass, Green, Spice
30	2-Acetyl pyrrole	1.14	1.000	2	–	–	2	Roasted, Bread, Cocoa
31	Acetophenone	0.98	0.002	0.01	8	32	8	Almonds, Floral
32	(3E,5E)-octa-3,5-dien-2-one	1.06	0.001	na	–	–	–	Fruit, Fatty
33	1-Octanol	0.99	0.003	0.022	8	–	–	Balsamic, Burnt Matches, Fat, Floral
34	Terpinolene	0.99	0.002	0.041	–	4	–	Pine, citrus
35	α-Naginatene	1.00	0.001	0.08	–	2	–	Caramel, Mint
36	Methyl benzoate	0.99	0.002	0.0015	–	128	–	Herb, Lettuce, Prune, Violet, Phenolic
37	Ethyl heptanoate	1.01	0.001	0.24	–	2	–	Brandy, Fruit, Wine
38	Nonanal	0.73	0.062	0.002	256	256	512	Fat, Floral, Green, Bean
39	Phenethyl alcohol	1.01	0.000	0.012	64	–	–	Fruit, Honey, Lilac, Rose
40	1,2,4,5-Tetramethylbenzene	1.10	0.002	0.087	4	4	–	Rancid sweetness
41	(2E,6E)-nona-2,6-dienal	1.01	0.001	0.0014	128	–	–	Fruit, Green
42	Ethyl benzoate	1.02	0.001	0.05	–	–	–	Camomile, Flower, Fruit
43	DL-Menthol	0.95	0.011	na	64	–	–	Mint, Cool
44	Naphthalene	0.99	0.006	0.3	–	–	–	Pungent
45	4'-Methylacetophenone	0.99	0.003	0.002	–	64	–	Almond, Floral, Sweet
46	Ethyl (*Z*)-oct-4-enoate	1.01	0.001	na	–	–	–	Fresh, Pineapple
47	Ethyl caprylate	1.01	0.002	0.022	32	64	64	Pineapple, Floral, Fruit
48	Decyl aldehyde	1.00	0.051	0.0026	64	32	–	Floral, Fried, Citrus, Aldehydic
49	β-Cyclocitral	0.99	0.001	0.019	32	16	16	Herbal, Rose, Tropical
50	2,6,6-Trimethyl-1-Cyclohexene-1-acetaldehyde	0.98	0.003	na	8	–	–	Camphorous, Berry, Soap
51	Citral	1.02	0.001	0.06	–	8	–	Cirtus, Strong Lemon
52	Ethyl 2-hydroxybenzoate	1.01	0.001	0.005	32	–	–	Sweet, Mint, Green
53	1-Phenylethyl propionate	1.02	0.001	na	–	–	–	Fresh, Floral, Fruit
54	Nonanoic acid	1.01	0.001	0.0016	512	–	–	Fat, Green, Sour
55	Ethyl nonanoate	0.97	0.007	0.009	32	64	8	Floral, Fruit
56	Gamma-Nonanolactone	0.98	0.003	0.0045	4	–	–	Coconut, Sweet
57	Alpha-Copaene	1.00	0.001	6	2	–	–	Honey, Woody
58	Ethyl caprate	0.87	0.012	0.0012	128	256	256	Fruit, Coconut
59	Alpha-Ionone	1.01	0.001	0.004	64	32	32	Violet, Woody
60	6,10-Dimethyl-5,9-undecadien-2-one	1.05	0.50	60	–	–	–	Fruit, Floral
61	3-Buten-2-one	1.02	0.001	0.00012	–	512	–	Floral, Woody, Violet
62	Ethyl Undecanoate	1.05	0.013	0.0003	–	256	32	Soap, Coconut, Cognac
63	Butylated Hydroxytoluene	1.02	0.001	na	–	8	–	Toasted Cereal, Phenolic
64	β-bisabolene	1.09	1.00	na	–	–	2	Woody, Balsamic
65	Delta-Cadinene	1.01	0.001	na	8	–	–	Woody, Herbal
66	Dihydroactinidiolide	0.95	0.017	na	8	4	2	Fruit
67	Ethyl laurate	0.82	0.99	0.002	128	128	64	Floral, Sweet, Waxy, Fruit
68	Pentadecanal	0.99	0.001	0.43	–	–	–	Waxy
69	Ethyl myristate	0.95	0.002	0.18	4	4	2	Violet, Sweet, Waxy,
70	Ethyl pentadecanoate	1.10	0.001	na	16	–	16	Honey, Sweet
71	Palmitic acid ethyl ester	0.84	0.77	2	–	–	–	Waxy, With little Creamy & Fruit
72	Ethyl Linoleate	0.82	0.003	0.45	–	–	–	Fatty, Fruit

1 Compound description reported from Scifinder (American Chemical Society) and description of FEMA flavor library. 2 na this compound data is not available. 3 The "-" mark indicates that the volatile compound was not sniffed. 4 Odor description reported from Flavor-Base 10 and description of FEMA flavor library, odor threshold mainly refers to the report by van Gemert et al. (2003) [29].

Table S3. the characterization of 3-(Methylthio)propionaldehyde (Methional).

Time (min)	Name	compare index	molecular weight	CAS numbers	NIST	NIST number	Type	Column and Detector
**8.049**	**Methional**	**72**	**104.03**	**003268-49-3**	**NIST17**	**5240**	**Sample**	**HP-5MS, 8890GC-5977MS**
**8.206**	**Methional**	**87**	**104.03**	**003268-49-3**	**NIST20**	**5323**	**standard**	

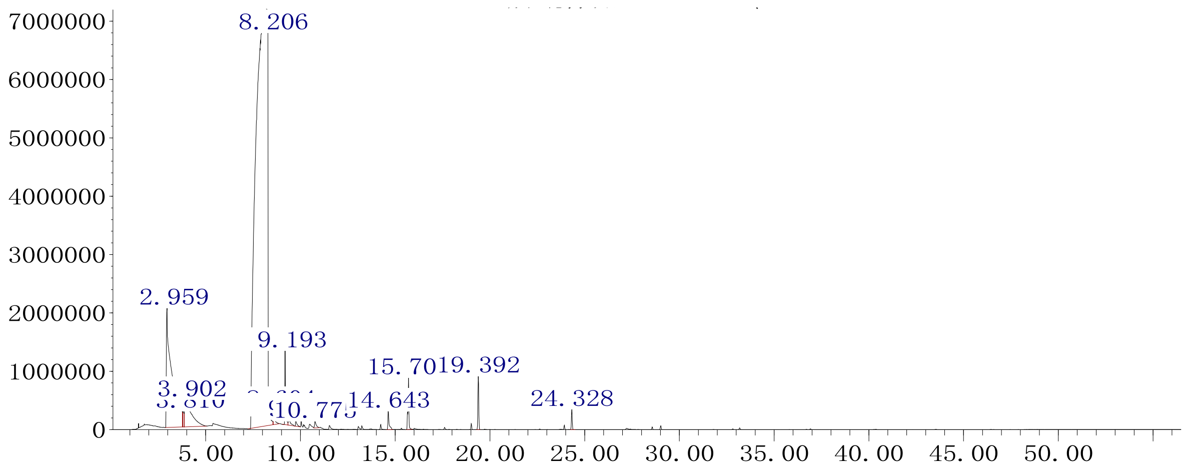

Figure Total ion current chromatogramof standard 3-(Methylthio)propionaldehyde (Methional)

Table S4. Structre of Aroma-active compounds identified by SPME-GC-O-MS and GC-IMS (based on OAV＞1).

No.	Compounds	Structure	FD			OAV			Perception threshold (mg/kg)	Odor series
			T1-1	T1-2	T1-3	T1-1	T1-2	T1-3		
	**Aldehydes**									
1	Isovaleraldehyde	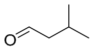	–	256	–	-^{a}	123±71^{b}	-^{a}	0.0001	6,12
2	2-Methylbutyraldehyde	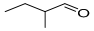	–	32	–	-^{a}	3.73±0.61^{b}	-^{a}	0.02	1,13
3	Hexanal	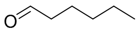	256	128	256	73.71±3.64^{a}	33.86±8.29^{b}	42.07±8.21^{b}	0.0014	2,4,6
4	Hex-2-enal	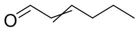	8	–	–	6.28±1.49^{b}	-^{a}	-^{a}	0.03	1,8
5	Trans-2-Hexenal	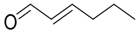	64	–	8	6.28±1.49^{b}	-^{a}	1.22±0.08^{c}	0.03	6,8
6	(Z)-4-Heptenal	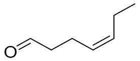	64	–	–	20.32±2.06^{b}	-^{a}	-^{a}	0.0034	2,8
7	3-(Methylthio)propionaldehyde	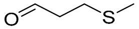	–	512	512	-^{a}	1012.7±192.06^{b}	1136.51±142.86^{b}	0.000063	7,11
8	Benzaldehyde	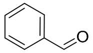	8	–	8	6.49±0.56^{b}	-^{a}	5.52±0.8^{b}	0.1	1,6,7,12,14
9	Phenylacetaldehyde	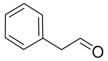	128	128	256	66.25±3.58^{c}	86.92±6.5^{b}	85.25±4.33^{b}	0.0012	5,6,10,12
10	(E)-2-Octenal	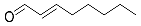	16	–	–	19.67±3.39^{b}	-^{a}	-^{a}	0.012	2,4,6,8,10
11	1-Nonanal	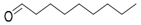	256	256	512	198.1±50.6^{b}	114.85±24.5^{b}	203.25±67.55^{b}	0.002	2,5,6,8
12	(2E,6E)-nona-2,6-dienal	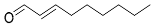	128	–	–	167.71±19.57^{b}	-^{a}	-^{a}	0.0014	6,8

No.	Compounds	Structure	FD			OAV			Perception threshold (mg/kg)	Odor series
			T1-1	T1-2	T1-3	T1-1	T1-2	T1-3		
13	Decyl aldehyde	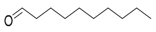	64	8	–	52.12±19.73b	19.35±5.81b	-a	0.0026	2,5,6
14	β-Cyclocitral	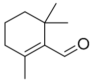	32	16	8	12.63±0.96[b]	4.27±0.36[c]	5.14±0.72[c]	0.0193	5,9,12
15	Citral	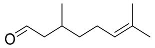	–	8	–	-[a]	1.19±0.15[b]	-[a]	0.06	6
16	Butanal	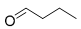	1	4	4	2.80±0.04[c]	7.84±0.15[a]	6.70±0.13[b]	0.0013	6，8，10
	**Alcohols**									
17	1-Penten-3-ol	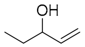	16	–	–	12.38±3.12[b]	-[a]	-[a]	0.01	2,4
18	1-Hexanol	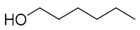	32	–	–	3.26±0.26[b]	-[a]	-[a]	0.034	5,9,12
19	1-Octanol	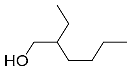	8	–	–	14.28±3.82[b]	-[a]	-[a]	0.022	2,3,5,7,15
20	Phenethyl alcohol	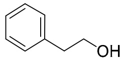	64	–	–	22.34±2.50[b]	-[a]	-[a]	0.012	5,6,12
	**Esters**									
21	Butyl acrylate	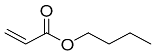	128	–	–	56.55±11.27[b]	-[a]	-[a]	0.00055	6
22	Ethyl valerate	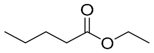	–	256	–	-[a]	101.72±19.83[b]	-[a]	0.00058	6,9
23	Ethyl Hexanoate	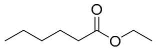	–	64	–	-[a]	42.13±6.77[b]	-[a]	0.003	6,12

No.	Compounds	Structure	FD			OAV			Perception threshold (mg/kg)	Odor series
			T1-1	T1-2	T1-3	T1-1	T1-2	T1-3		
24	Methyl benzoate	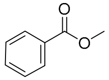	–	128	–	-^{a}	61.53±15.53^{b}	-^{a}	0.0015	5,6,8,9
25	Ethyl caprylate	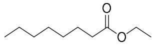	4	8	64	3.06±0.48^{c}	30.72±6.67^{b}	37.83±6.74^{b}	0.022	5,6
26	Ethyl 2-hydroxybenzoate	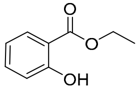	32	–	–	14.84±1.4^{b}	-^{a}	-^{a}	0.005	8,12
27	Ethyl nonanoate	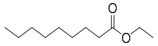	32	64	8	6.78±0.84^{c}	21.92±4.99^{b}	8.11±1.84^{c}	0.009	5,6
28	Gamma-Nonanolactone	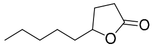	4	–	–	5.91±1.6^{b}	-^{a}	-^{a}	0.0045	6,12
29	Ethyl caprate	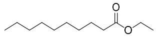	128	256	256	87.25±10.25^{b}	712±245.75^{b}	565.25±205.83^{b}	0.0012	1,6
30	Ethyl Undecanoate	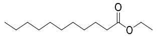	–	512	128	-^{a}	988.33±397.33^{b}	111.67±18.67^{b}	0.0003	1,2,6,16
31	Ethyl laurate	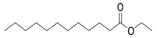	128	128	64	205.15±60.7^{b}	205.55±78.35^{b}	106.85±30.45^{b}	0.002	5,6,8,12,15
32	**Ketones**<br>2-Octanone	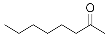	8	4	–	2.81±0.14^{b}	0.58±0.08^{c}	-^{a}	0.23	2,5,11
33	Isophorone	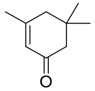	–	–	32	-^{a}	-^{a}	55.18±1.88^{b}	0.0017	8,9

No.	Compounds	Structure	FD			OAV			Perception threshold (mg/kg)	Odor series
			T1-1	T1-2	T1-3	T1-1	T1-2	T1-3		
34	Acetophenone	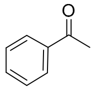	8	32	8	5.18±0.7[c]	27.44±5.2[b]	8.64±0.6[c]	0.01	1,5
35	4'-Methylacetophenone	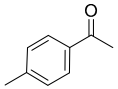	–	64	–	-[a]	24.85±6.55b	-[a]	0.002	1,5,12
36	α-Ionone	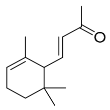	64	32	32	124.53±6.98[a]	22.6±2.33[c]	24.2±1.38[c]	0.004	3,5
37	3-Buten-2-one	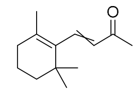	–	512	–	-[a]	962.5±41.67[c]	-[a]	0.00012	3,5
38	3-Hydroxy-2-Butanone	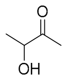	4	1	1	2.43±0.08[a]	0.44±0.01[b]	0.42±0.01[b]	0.014	2,8
39	**Alkenes**<br>(+)-Dipentene	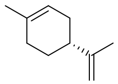	16	32	–	4.11±1.09[c]	15.61±2.08[b]	-[a]	0.045	6,8
40	Terpinolene	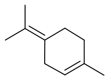	–<br>–	4	–	-[a]	1.65±0.37[b]	-[a]	0.041	3,6
41	**Aromatic compounds**<br>1,2,4,5-Tetramethylbenzene	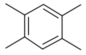	4	4	–	0.94±0.16[c]	1.72±0.11[b]	-[a]	0.087	12,16
42	**Acids**<br>Nonanoic acid	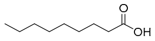	512	–	–	269.31±14.75[b]	-[a]	-[a]	0.0016	2,8,16

No.	Compounds	Structure	FD			OAV			Perception threshold (mg/kg)	Odor series
			T1-1	T1-2	T1-3	T1-1	T1-2	T1-3		
	**Nitrogenous compounds**									
**43**	2-Acetyl-1-pyrroline	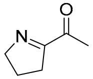	**256**	**512**	**512**	**718.01±27.28[b]**	**1065.24±17.93[a]**	**1080.06±13.22[a]**	**0.00003**	**7,12**
